# Emerging technology has a brilliant future: the CRISPR-Cas system for senescence, inflammation, and cartilage repair in osteoarthritis

**DOI:** 10.1186/s11658-024-00581-x

**Published:** 2024-05-02

**Authors:** Shicheng Jia, Rongji Liang, Jiayou Chen, Shuai Liao, Jianjing Lin, Wei Li

**Affiliations:** 1 Department of Sports Medicine and Rehabilitation, Peking University Shenzhen Hospital, Shenzhen, 518036 China; 2 Shantou University Medical College, Shantou, 515041 China; 3 Department of Bone and Joint, Peking University Shenzhen Hospital, Shenzhen, 518036 China; 4 Shenzhen University School of Medicine, Shenzhen, 518060 China

**Keywords:** CRISPR-Cas system, Osteoarthritis, Cellular senescence, Inflammation, Cartilage repair

## Abstract

Osteoarthritis (OA), known as one of the most common types of aseptic inflammation of the musculoskeletal system, is characterized by chronic pain and whole-joint lesions. With cellular and molecular changes including senescence, inflammatory alterations, and subsequent cartilage defects, OA eventually leads to a series of adverse outcomes such as pain and disability. CRISPR-Cas-related technology has been proposed and explored as a gene therapy, offering potential gene-editing tools that are in the spotlight. Considering the genetic and multigene regulatory mechanisms of OA, we systematically review current studies on CRISPR-Cas technology for improving OA in terms of senescence, inflammation, and cartilage damage and summarize various strategies for delivering CRISPR products, hoping to provide a new perspective for the treatment of OA by taking advantage of CRISPR technology.

## Introduction

Osteoarthritis (OA), known as one of the most common types of aseptic inflammation of the musculoskeletal system, is characterized by defects of hyaline cartilage, synovial inflammation, subchondral bone loss, and tissue hypertrophy [1]. Its main clinical symptoms are chronic pain and whole-joint lesions, and eventually disability [2, 3]. The prevalence of OA has increased steadily because of obesity, trauma, and the aging population [4]. Despite its high prevalence, there are no drugs that can inhibit the progression and eliminate symptoms of OA absolutely, and medications recommended by guidelines usually have dose-dependent toxicity [5, 6]. Considering that OA has a high gene-related possibility, estimated at 40–60%, gene therapy may be able to provide more valuable ideas for the treatment of OA [7].

Currently, molecular biology, genetics, and genomics are facing a historic opportunity. Since clustered regularly interspaced short palindromic repeats (CRISPR) was discovered in the 1980s, CRISPR and the CRISPR-associated system (Cas) have been rapidly developed into a third generation of gene-editing tools. Essentially, CRISPR is a defensive sequence within the prokaryotic genome, and Cas represents genes located on the CRISPR locus nearby [8]. In a broad sense, the core concepts of the CRISPR-Cas system are the CRISPR locus, the related Cas genes, and the RNA-guided adaptive immune system encoded by related genes [9, 10]. As a type of RNA sequence, the CRISPR locus contains spacers originating from bacteriophages and extrachromosomal elements and is separated by sequences that are short, repeat, and can encode small nonmessenger RNA [11]. Generally, it can be divided into a leader region, repeat region, and spacer region. CRISPR RNA (crRNA) derives from precursor CRISPR transcription through processing of nucleic acid endonuclease; it can pair with complementary target sequences by the spacer at the 5′ end and trigger specific disruption of an invading sequence by Cas nuclease from Cas genes [12]. Thus, the decisive characteristic of the CRISP-Cas system is the effectors composed of crRNAs and Cas proteins, with the ability to recognize and disturb targeted sequences [13, 14]. Compared with conventional tools such as zinc finger nucleases, recombinases, transcription activator-like effector nucleases, and restriction enzymes, the CRISPR-Cas system offers more advantages for use in OA therapy [15]. It has a more powerful ability to regulate gene expression and genome sequence, more precise insertion, knockout, and edition of targeted genes, and inducing more phenotypic protein [16]. Improved CRISPR-Cas systems can produce specific sequences rapidly and be used easily, promoting their application within gene therapy [11]. However, the application of the CRISPR-Cas system requires clarification of the molecular biology and genomic mechanisms to identify optimal editing sites.

Although OA is a complex, multigenetic, and multitissue degenerative disease, researchers have explored its pathogenesis and structure degeneration comprehensively [17]. Senescence, inflammatory alterations, and the corresponding regulation of genes, proteins, and signaling pathways are key factors that induce the development of OA [1, 18, 19]. Once pathological signaling pathways are activated, changes such as excessive apoptosis [20], autophagy [21], pyroptosis [22], hypertrophy [23], disturbance of metabolism [24], and abnormal differentiation [25] occur among chondrocytes. Combined with the influence of inflammatory mediators (e.g., proinflammatory cytokines), processes of subchondral bone sclerosis, degeneration of extracellular matrix, production of reactive oxygen species, and destruction of collagen are initiated [1, 26–28], and OA will develop and progress continuously, causing cartilage defects. Thus, OA is regulated by multiple signaling pathways and results from deterioration of cell fate and the interaction of tissues such as cartilage and synovium. The signaling pathways and corresponding molecular products involved in these processes offer potential targets for the treatment of OA, enabling the use of gene-editing therapies, especially with the CRISPR-Cas system, as potential tools for OA treatment.

In this review, we summarize the structure, mechanism, and function of the CRISPR-Cas system. Besides, we provide recent insights into OA gene therapy from the aspects of cellular senescence, inflammation, and cartilage repair. The inclusion of up-to-date research is highlighted to summarize and predict potential developments. We also present reviews of and insights into tools for delivering the CRISPR-Cas system.

## Overview of current therapeutic strategies for osteoarthritis

Both primary OA (caused by the degeneration of bone and cartilage tissue) and secondary OA (caused by trauma, inflammation, fracture, etc.) have a similar pathological mechanism: Changes in molecules and the ECM increase the level of inflammatory cytokines and enzymes, which destroy cartilage structure and disturb the process of cartilage repair. Thus, cartilage will disappear, and the resulting direct friction between bones causes pain and even disability [29]. This dictates that the treatment of OA ultimately comes down to the control of inflammation and the repair of damaged cartilage.

Until now, conventional strategies for preventing exacerbation of OA have been primary therapies such as weight control, exercise control, and trauma prevention [30]. Other conventional therapy aims to relieve the symptoms. For example, nonsteroidal antiinflammatory drugs (NSAIDs) are often used to reduce the pain of patients [31]. Besides NSAIDs, chondroitin sulfate is generally recognized as an effective nutritional factor that benefits cartilage. In addition to oral medications, intraarticular injections of lubricating agents, such as sodium hyaluronate, can reduce the increased interbone friction that occurs after injuries to articular cartilage, thereby relieving symptoms [29, 32]. For patients with severe OA, surgery is the last choice of treatment [33]. Effective strategies include arthroscopic debridement, osteotomy, and ultimately arthroplasty. However, they carry the risks of iatrogenic injury, periprosthetic infection, and eventual joint revision [34–36].

To strengthen the effect of nonsurgical treatment and avoid the side effects and trauma of surgical treatment, as well as to maximize the fundamental solution for cartilage defects and other problems brought about by OA, cell therapies and gene therapies (sometimes combined) have been proposed. Culturing autologous chondrocytes in vitro and injecting them into joints in the form of articular cavity injections for cartilage repair is a widely recognized option in recent years [37–39]. Meanwhile, owing to their multispectral differentiation, immunomodulatory function, low immunogenicity, and self-renewal ability, MSCs are becoming an emerging therapy that is being focused on to avoid passaging-induced chondrocyte dedifferentiation while taking full advantage of their important roles in tissue regeneration and repair in response to cartilage deficits caused by OA [40, 41]. Additionally, extracellular vesicles (EVs) secreted by MSCs have also been shown to promote ECM synthesis and cartilage repair [42]. Their therapeutic function is mainly achieved by effectively regulating the expression levels of inflammatory genes, catabolic genes and synthetic genes, and immunomodulation of cells and microenvironment within the OA environment [43–45]. However, all such explorations must confront the dilemma of whether chondrocytes and MSCs can effectively colonize, proliferate, and form mature cartilage tissue in a difficult OA environment. Furthermore, the cost of cell therapy, the risk of additional surgery required to extract the cells, and the safety of clinical translation are all issues that should be balanced.

Gene therapies are designed to regulate the expression of damaged genes by regulating genes (alone, or in combination with cellular therapies) to achieve the goal of superiority over cellular therapies or conventional therapeutic molecules. As knowledge of OA continues to grow, gene therapy is advancing with it. The most accepted gene-related therapeutic regimen is intraarticular delivery of various gene enhancers or inhibitors. For example, targeting IL-1β, which is involved in the pathological mechanism of OA, lowering its expression level, or blocking its receptor are considered effective therapeutic options. Based on this, IL-1 receptor antagonists are one of the most promising gene therapies; they can inhibit multiple signal transduction on the corresponding signaling pathway and effectively reverse disease progression in OA models [29]. Another idea is to highly express genes that promote cartilage synthesis in vivo. It has been shown that the use of insulin-like growth factor to promote proteoglycan synthesis in rabbit knee joints was effective for stimulating matrix synthesis in OA joints [46]. And related studies targeting SOX9, FGF-2, and hyaluronan synthase 2 have shown therapeutic effects on OA [47–49]. Currently, theories based on various types of RNA dysregulation leading to OA have greatly facilitated the development of RNA-related gene therapies [29]. Several studies have reported that intraarticular injections of nonviral or viral vector-loaded miRNAs ameliorate pathological changes in OA [50–52]. And using small interfering RNAs to specifically inhibit expression of MMP13, which plays a major role in OA progression, has also been shown to be an effective gene therapy option [53]. It should be noted that miRNAs are susceptible to off-target effects, whereas siRNAs are more susceptible to degradation, making their effects relatively unstable. In addition, the effects of utilizing RNAs are largely dependent on their effectiveness and specificity. These characteristics limit the application of noncoding RNA-based gene therapy [54]. In contrast, CRISPR-based approaches have shown greater potential owing to their high efficiency, weaker off-target effects, and versatility, which points to a new direction for gene therapy [7, 16].

## Structure, mechanism, and function of the CRISPR-Cas system

According to the current CRISPR-Cas loci and mechanisms, existing CRISPR-Cas systems can be divided into two classes [55]. Class I includes type I and III systems, composed of heteromeric multiprotein effectors, and carry out biological function through a large multi-Cas protein complex [14, 56]. Conversely, type II, V, and VI systems belong to class II and are frequently used because they form a single multidomain effector [57, 58].

**Fig. 1:**
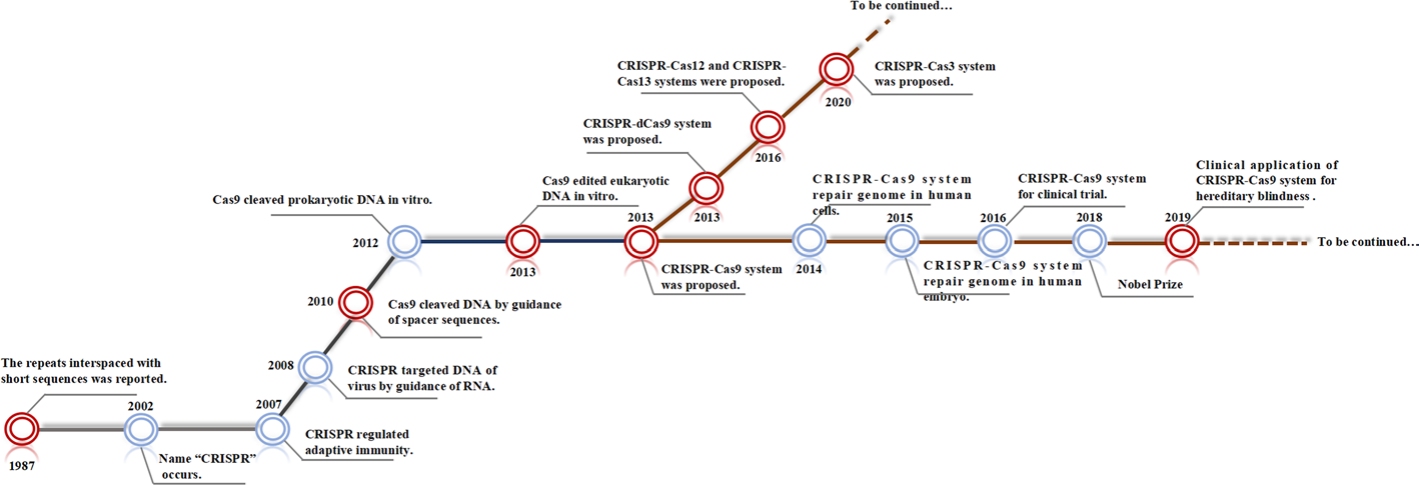
Timeline and overview of development of the CRISPR-Cas system

CRISPR-Cas9, which recognizes and cleaves double-strand DNA (dsDNA) by employing single DNA endonuclease, is the most utilized tool that benefits from the specificity and codability of RNA [59]. It is composed of guide RNA (gRNA) and Cas9 proteins with nucleic acid endonuclease function; the gRNA guides Cas protein to target sites, where double-strand DNA is ruptured through the influence of the Cas protein, and is then repaired by the endogenous pathway [60–64]. The realization of this process relies on high GC proto-spacer adjacent motif (PAM, a noncoding short fragment on crRNA), trans-activating RNA (tracrRNA), crRNA, and Cas9. gRNA is synthesized by a combination of crRNA and tracrRNA, where the former identifies targeted sequences of DNA and the latter combines Cas9 protein [57]. Cas9 has a recognition lobe (REC) containing bridge helix and three helical domains, and a nuclease lobe (NUC) with a Topo domain, a HNH domain, a C-terminal domain (CTD), and a split RuvC domain. The RuvC domain is activated to cleave DNA strands that are opposite to complementary strands (i.e., nontargeted DNA), and the HNH domain is activated to cleave DNA strands that are complementary with crRNA (i.e., targeted DNA) [65]. Subsequently, Doudna and Charpentier fused crRNA and tracrRNA into a single RNA and named it single-guide RNA (sgRNA) [66]. The improved CRISPR-Cas9 system provided revolutionary progress for gene therapy (Fig. 1 shows the timeline of the progress of the CRISPR-Cas system).

The mechanism of action of the CRISPR-Cas9 system can be summarized as follows: Cas9 cuts the sequences on the targeted DNA with the guidance of sgRNA, which produces double-strand break (DSB). The DNA will then be repaired as in autonomous cells via a process that involves nonhomologous end joining (NHEJ) and homologous recombination (HR) [12]. NHEJ directly shortens the distance between the ends of broken strands and then rejoins the broken strands with the help of DNA ligase, whereas HR prefers DNA exchange between homologous chromosome regions [67]. NHEJ and HR have different characteristics, and each has its own advantages and disadvantages. A specific comparison between NHEJ and HR is presented in Table 1 [68–72].

**Table 1:** A comparison between NHEJ and HR

	NHEJ	HR
Prerequisites for repairing	No need for template	Homologous DNA template
Phase for repairing	All of the cell cycle	Most efficient for late S and G2 phases
Advantages for repairing	High frequency	Allows specific point mutations and sequence insertions
Disadvantages	Easier for random insertions and deletions	Low frequency

Besides Cas9, researchers have explored many new Cas proteins to develop favorable type II CRISPR-Cas systems. For instance, Qi et al. introduced dead-Cas9 (dCas9) in 2013 [73]. Mutations in the RuvC and HNH domains on dCas9 cause Cas proteins to have only targeting function and lose their nuclease function. dCas acts as a tool for precise targeting and can form fusion proteins with other effectors [73, 74]. This allows the CRISPR-dCas9 system to target and regulate gene expression without causing DNA damage. Another explored approach is the CRISPR-Cas12 system, with 11 subtypes labeled from a to k [75]. Cas12a, 12b, and 12f are commonly used. Cas12a prefers recognizing a high content of T nucleotide PAM, rather than a high content of GC like Cas9. It functions through a single RuvC domain and is guided by a single crRNA, whereas Cas12b is guided by crRNA and tracrRNA [75, 76]. In addition to the routine function of Cas proteins to cleave dsDNA, Cas12a, 12b, and Cas12f have the ability to trans-cleave single-strand DNA (ssDNA) without dependence on PAM. Thus, full utilization of the ssDANase activity of Cas12 can provide sensitive, specific, and rapid new solutions for gene therapy and molecular diagnostics [77–79]. In contrast, the CRISPR-Cas13 system is a type VI system and has been identified as a potential tool targeting RNA [80]. Although the CRISPR-Cas13 system has been explored and divided into seven subtypes (a, b1, b2, c, d, X, and Y), all the types have similar single effector Cas13 proteins with two different RNase activities: one to target, cleave, and generate the RNA sequence, and the other to preprocess crRNA [81–83]. In summary, numerous CRISPR-Cas13 systems have been developed and applied in RNA degradation, live imaging, nucleic acid detection, and base edition [84], and further progress on the CRISPR-Cas13 system will provide a new gene therapy and gene-editing platform for OA.

## Biological and biomaterial-related delivery systems for the CRISPR-Cas system

Although CRISPR-Cas has been regarded as a revolutionary technology for gene editing and transcriptional regulation since 2012 because of its unparalleled advantages such as precise editing of multiple targets, rapid generation of mutants, and the possibility of designing single guide RNAs (sgRNAs) [85–87], its components must be delivered under stringent conditions by special tools. Strategies to deliver CRISPR-Cas systems efficiently and safely have gradually become an issue that must be solved and innovated. The ideal delivery system for CRISPR components should be efficient, highly safe, stable, and nontoxic [88]. Conventional viral vectors are limited by oncogenicity, immunogenicity, compositional constraints, mass production efficiency, and Cas expression lifespan, while for nonviral vectors, one needs to address issues such as rapid clearance, toxicity, biocompatibility, and release of active ingredients [87, 89]. In addition, a variety of abiotic delivery options are worth considering Several current delivery systems are summarized in Table 2.

**Table 2:** Some current delivery systems for the CRISPR-Cas system

Delivery system	Type of CRISPR-Cas system	Experimental model	Effects and comments	Ref.
AAVs	Cpf1	Primary human hepatocytes	The mutation rates were estimated at around 12% of insertion/deletion (indel), with transduced human hepatocytes at 2 weeks after transduction	[111]
Baculovirus	dCas9-VPR and gRNA	Rat adipose stem cells	Successfully induced gene transfection and achieved efficient gene editing	[112]
Dendrimers	Cas9 mRNA, sgRNA, and donor DNA	HEK293 B/GFP cells	By optimizing the system for simultaneous delivery of Cas9 mRNA, sgRNA, and donor DNA, the delivery system via dendritic lipid nanoparticles enables editing of more than 91% of cells, achieving integrated, concise, and efficient gene editing	[113]
	Cas9 RNP	293 T cells	Owing to the presence of boric acid, the vectors can bind to differently charged proteins simultaneously, effectively maintaining the activity of the delivered Cas9 and enabling efficient CRISPR-Cas9 editing	[114]
Lipid nanoparticles	Cas9 mRNA and sgRNAs	HEK293/GFP cells	The LNPs enabled up to ~ 80% gene editing in vivo	[115]
	Cas9 mRNA and sgRNA	Duchenne muscular dystrophy mice model	The LNPs induces stable genomic exon skipping and have shown promising therapeutic effects in mice. In addition, LNPs can target multiple muscle groups and are characterized by repeated administration and low immunogenicity	[116]
Micelles	Cas9 mRNA and sgRNA	Parenchymal cells in the mouse brain	Co-encapsulation of sgRNA with Cas9 mRNA in micelles prevents release of sgRNA upon dilution, thereby increasing the tolerance of sgRNA to enzymatic degradation	[117]

*Viral delivery systems* have the abilities to integrate into the host genome, produce sustained effects, and deliver compositions efficiently [90]. Among the variety of viral vectors, adenoviruses, adeno-associated viruses (AAVs), and lentiviruses play an important role in CRISPR-Cas-based genome-editing therapies and have been widely used in clinical models and trials [91]. As an 80–100-nm double-stranded DNA virus, adenovirus itself can carry up to 8 kb of exogenous DNA and enhance transfection of the CRISPR-Cas system through additional targeting signals [92]. In addition, adenoviruses can infect both dividing and nondividing cells and effectively minimize off-target effects and unintended mutations [91, 92]. In contrast, ideal AAVs have a transmission capacity of 4.1–4.9 kb and recombinant AAV must also contain articular regulatory elements for gene expression, so even though the vectors themselves may be much larger than the size of the CRISPR-Cas system, the packaging efficiency is severely reduced, and they cannot be used for extensive gene regulation [90, 93]. Another serious problem is that the presence of neutralizing antibodies against AAV in patients previously infected with AAV significantly reduces the transfection efficiency [94]. The property of AAV to promote long-term Cas expression also increases the risk of off-target effects [95]. However, AAV is often used as an in vivo transfection system and exhibits tropism for different organs depending on the serotype and phenotype [90]. In general, the combination of capsid regulation and genomic regulation provides AAV serotype vectors that reduce the affinity of neutralizing antibodies for drug-resistant reactions and increase the transfection efficiency [95]. The intra-articular injection of adeno-associated virus, which expressed CRISPR/Cas9 components to target genes encoding MMP13, IL-1β, and NGF, successfully achieved gene editing in a surgically induced OA mouse model [96]. Compared with adenovirus and AAV, lentivirus, as a type of retrovirus, has low cytotoxicity and weak immunogenicity, with little side effects on transfected cells [90, 97]. Although it also faces difficulties in off-target effects due to continuous Cas9 expression and high-precision genome editing, the use of selective integrase-deficient lentiviral vectors generated by integrase modification significantly reduces the risk of unintended mutations [98, 99]. For all viral vectors, the use of glycoproteins for viral surface wrapping modification, or deletion of promoters or enhancers with terminal repetitive sequences to avoid the activation of relevant genes, are effective methods to improve the safety of transfection and delivery of viral vectors [90].

*Nanoparticle delivery systems* have revolutionized the field of genome editing in the context of the rapid development of synthetic vectors, biomaterials, and cell engineering. Nonviral vectors are less limited by packaging capacity and minimize immunogenicity [100]. At the same time, Cas delivered by nonviral vectors tends to be expressed transiently, reducing the probability of insertion mutagenesis and the risk of nuclease-induced off-target effects [100, 101]. Lipid nanoparticles (LNP) artificially polymerized molecular nanoparticles have been widely used and are recognized as mainstream [16, 90, 101]. Lipid nanoparticles are essentially amphiphilic, bilayer vesicle-like carriers composed of various hydrophobic and hydrophilic molecules that mimic cell membranes [102]. Owing to their efficient delivery ability and good biocompatibility, they have promising applications in the delivery field. LNPs are characterized as a targeted delivery system with cargo monitoring and reduced toxicity [103]. In particular, the ionic and polar head of cationic liposomes allows unstable nucleic acids with anions to better cross the cell membrane, making them highly sought after for gene delivery, especially nuclear transport [90, 101, 102]. Liposomes prepared by Han et al. using microfluidics can increase the encapsulation of terminal sgRNA up to 85% [104]. Based on the advantages of high bioavailability, biocompatibility, long lifetime in blood circulation, and degradability of polymeric materials, the use of protein cores and polymeric encapsulation of CRISPR-Cas system to form a nanodelivery system for effective gene delivery is considered to have good development prospects [105, 106]. Although artificial polymeric molecular nanoparticles could offer a new delivery system of gene therapy, it is still unclear whether they can realize their advantages in the circulatory system, as local injection is often considered for the treatment of OA.

*Extracellular vesicles* as the delivery system for genetic components has received increasing academic attention [88]. As functional materials secreted by various natural cells under different external or internal conditions, EVs can regulate biological processes by themselves while offering effective delivery, targeted delivery, and biocompatibility through their phospholipid bilayer membranes and high-level messenger molecules on the surface [107–109]. Therefore, both artificially modified and natural EVs are reliable and are expected to deliver CRISPR-related components with high safety. Hybrid exosomes formed by membrane fusion of chondrocyte-targeting exosomes with liposomes entered the deep region of the cartilage matrix in OA rats, delivering the plasmid Cas9 sgMMP-13 to chondrocytes [110]. However, accurate delivery of components via EVs is problematic owing to various types of interference. Delivery of EVs based on the CRISPR-Cas systems is still in its infancy, and multiple issues need to be addressed: (1) the standardization and engineering of EV preparation, (2) the uncertain interactions, pharmacokinetics, and biodistribution of EVs and intrinsic CRISPR components, (3) clarification of methods for administration of EVs, (4) bioregulatory functions due to their own bioregulatory functions, so one cannot ignore homogenization of EV delivery systems for different diseases and the trade-off between generalizing the types of EVs for broad categories of diseases or targeting development for each different disease, and (5) the need to consider organelle-specific EVs as a future research direction.

**Fig. 2:**
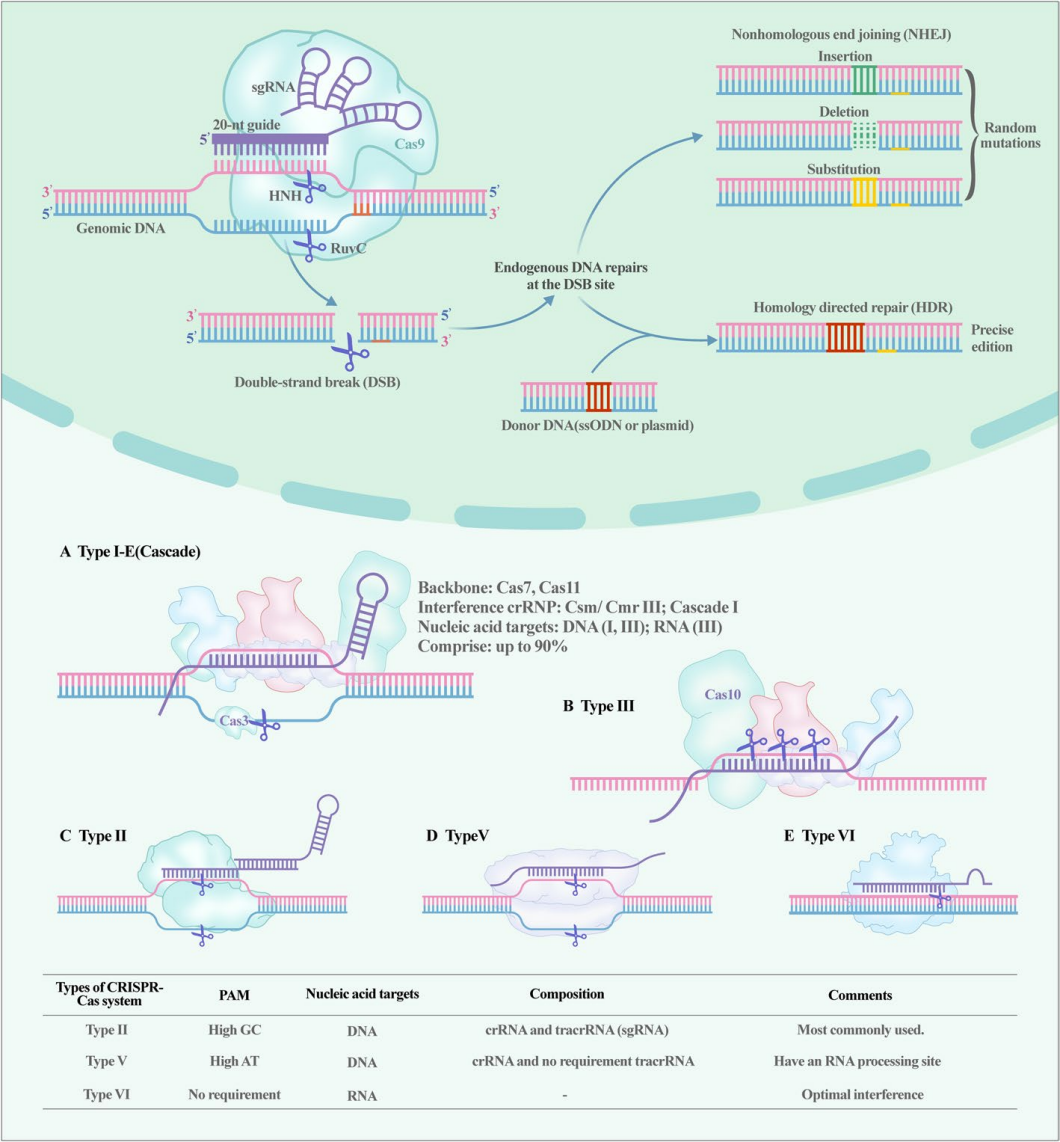
The mechanism of the classical CRISPR-Cas system and the classification of CRISPR-Cas systems. CRISPR-Cas9 shears through different structural domains on the Cas9 protein and repairs the sheared DNA by both NHEJ and HDR to accomplish gene editing. In turn, CRISPR-Cas is divided into different kinds according to the Cas

With the identification of structures, exploration of mechanisms, and development of platforms (Fig. 2), the CRISPR-Cas system has become an emerging technology that is receiving more attention in the gene therapy field. The combined application of different CRISPR-Cas systems provides the possibility for various gene-editing strategies. In the OA gene therapy field, this revolutionary technology has sufficient potential for diagnosis, reversing cellular senescence, improving inflammation, and promoting cartilage repair.

## Application of the CRISPR-Cas system for cellular senescence in the process of OA

Cellular senescence, known as a key risk factor in OA, is caused by multiple physical or pathological processes such as DNA damage, telomere shortening, oxidative stress, mitochondrial dysfunction, and sustained cytokine activation [118]. Apoptotic resistance, degeneration of extracellular matrix (ECM), secretion of proinflammatory molecules, and permanent arrest of proliferation are the common characteristics of senescence among various cellular types, being identified as the senescence-associated secretory phenotype (SASP) [119]. The accumulation of senescent nonreplicable chondrocytes will trigger inflammatory pathways, affect oxidative stress, inhibit energy metabolism in mitochondria, and destroy the balance between synthesis and elimination within cartilage homeostasis [120–123]. Preclinical studies have proved that removing the SASP through multiple gene-editing tools can attenuate the process of OA [124]. As an emerging gene-editing tool, CRISPR-Cas technology offers the possibility of effective validation of potentially relevant pathways and reversing cellular senescence phenotypes more efficiently and precisely.

Common senescence-related genes include telomerase-related genes that maintain chromosome stability and preserve telomere length [125], fibroblast growth factor (FGF) family genes that inhibit cellular senescence, oxidative stress, stem cell failure, and promotes autophagy through multiple signaling pathways (e.g., insulin/IGF-1, WNT, p53/p21, and forkhead box) [126, 127], forkhead box subgroup O (FOXO) family genes targeting oxidative stress, DNA damage, autophagy, and metabolism [128], SIRT family genes that affect the stability of genome, chronic inflammation, homeostasis of energy, metabolism, mitochondrial signaling pathways, and interactions with multiple other signaling pathways [129–132], vascular endothelial growth factor (VEGF) pathway for vessel formation [133], etc. Since senescence-related genes have been extensively studied, chondrocyte-associated senescence genes that promote OA progression are gradually being validated. Recent studies have shown that senescent chondrocytes during OA progression have two robust endophenotypes. One is endotype-1 with high expression of forkhead box protein O4 (FOXO4), cyclin-dependent kinase inhibitor 1B (CDKN1B), and RB transcriptional corepressor like 2 (RBL2), while the other is endotype-2 with potential therapeutic pathways of vascular endothelial growth factor (VEGF) C and SASP [134]. The CRISPR-Cas system plays an important role in exploring and validating such potential pathways and therapeutic targets. Yes-associated protein (YAP), known as an actor in the Hippo signaling pathway, plays a key role in cartilage homeostasis and cellular senescence [135]. Regulation of its expression will affect the integrity of the nuclear envelope, the transduction of cGAS-STING signals, and the formation of the SASP [136]. Fu et al. delivered a CRISPR-Cas9 system via lentivirus to knockout YAP in mice, verified its role in promoting the development of OA, and revealed the role of the YAP/FOXD1 axis in regulating cellular senescence as one of the major molecular mechanisms for OA progression [137]. The same protocol for exploring target genes was used to discover and validate the CBX4 gene by Liu et al. They utilized a CRISPR-Cas system to construct CBX4 knockout human mesenchymal stem cell (hMSC) models and found that deficiency of CBX4 leads to cellular senescence, whereas its overexpression alleviates cellular senescence and subsequent osteoarthritis through maintaining nucleolar homeostasis [138]. Meanwhile, Jing et al. added to the lack of genomic screening studies based on the CRISPR-Cas system by constructing a synergistic activation mediator (SAM) using CRISPR-based activation (CRISPRa) technology to screen for OA progression via relevant aging genes. The results showed that SRY-Box transcription factor 5 (SOX5) can activate age-protective genes such as high-mobility group box 2 (HMGB2) and attenuate cellular senescence by triggering epigenetic and transcriptional remodeling. In a subsequent validation phase, they found that delivering SOX5 through lentivirus attenuated age-dependent OA in aged mice [139].

In addition to being used as a detected technical tool for potential sites, gene therapies based on the CRISPR-Cas system for different endophenotypes and corresponding gene, phenotypes, and signaling cascades have great promise. Conventional gene therapy for cellular senescence commonly means the introduction of exogenous complementary cDNAs into target tissues and cells to repair genes that have become defective [140]. With the development of the CRISPR-Cas system, gene replacement, polygene editing, and epigenetic modification therapy have become possible strategies to slow or inhibit aging, which cannot be achieved by conventional gene therapy. In the application of gene knockout, CRISPR/Cas technology eliminates the laborious process of synthesizing and assembling protein modules with specific DNA recognition ability. Moreover, compared with TALEN and ZFN technologies, the design and synthesis of gRNA in CRISPR/Cas require significantly less effort, while exhibiting lower toxicity than ZFN technology [141–143]. The aforementioned advantages have also been observed in the regulation of cellular senescence. By mimicking a similar mechanism of disease requiring wound healing, Varela-Eirín et al. used the CRISPR-Cas9 system to specifically downregulate the expression of the gap junction channel protein connexin 43 (Cx43), which reduced the nuclear translocation of Twist-1 caused by the Cx43-mediated increase in gap junctional intercellular communication (GJIC) and inhibited the formation of SASPs through the downregulation of p53, p16INK4a, and NF-κB to retard chondrocyte senescence and tissue remodeling [144]. As the influences of senescence signaling pathways do not exist in isolation owing to the interaction between multiple pathological processes such as inflammatory factor release and excessive reactive oxygen species (ROS) formation, CRISPR-Cas system gene therapy solely targeting senescence is not fully developed at present, and the core direction of use remains the exploration of possible and potential genes. Unlike the clearly defined inflammatory genes, genetic disease genes, or cancer genes in the common use scenarios of the CRISPR-Cas system, modifications of specific genes may lead to serious side effects or adverse reactions due to the complex signaling cascade of the senescent genes and the unclear mechanisms. Only senescence genes that have been identified after enough bioinformatics analyses, gene sequencing, and functional tests make clear sense for treatment using the CRISPR-Cas system.

## Application of the CRISPR-Cas system for inflammation in the process of OA

Inflammation in the cartilage and synovial microenvironment has been recognized as a key factor in the progression of OA since the discovery of abnormally high levels of inflammatory plasma proteins in the blood and joint fluids of OA patients in 1959 [145]. High levels of complements, plasma proteins, inflammatory mediators, and cytokines are among the key features of OA [146]. For example, interleukin-1β (IL-1β), which is produced by chondrocytes, leukocytes, osteoblasts, and synoviocytes, can bind to IL-1 receptor (IL-1R) and activate transcription factors through the NF-κB and MAPK signaling pathways to regulate the inflammatory response, leading to the production of inflammatory mediators such as COX-2, PGE2, and NO and accelerating OA progression [147]. Additionally, tumor necrosis factor-α (TNF-α) is one of the most important inflammatory factors that stimulate inflammation in OA. By regulation of pathways such as NF-KB and PI3K/Akt, it stimulates the production of matrix metalloproteinase (MMP) -1, MMP-3, and MMP13 by cartilage, synovium, and subchondral bone layer-associated cells to break down cartilage collagen [147–149]. As a key inflammatory mediator that can synergize with TNF-α, IL-6 initiates signaling cascades through the regulation of MAPK, SATA3, ERK, and other signaling pathways to promote OA progression [150, 151]. In brief, different inflammatory mediators have corresponding regulatory pathways, and genetic modulation of any targets on the pathway by using CRISPR/Cas system-related techniques has the potential to significantly affect the final OA progression. The multiple inflammation-related pathways are summarized in Table 3 [24, 152–218]. Nowadays, as the implementation and development of disease-modifying OA drugs (DMOADs) are subject to a series of limitations [219], it is of great significance to conduct CRISPR-based targeted therapy to target inflammatory mediators and related pathways during the progression of OA.

**Table 3:** Inflammation-related signal pathways in the progression of OA

Pathway	Related genes	Inflammatory mediators	Sites of functions	Pathological mechanisms	Refs.
AMPK pathway	ERK1/2	IL-1β, IL-6, LIF, MMP-1 MMP-3, MMP-13, ADAMTS-4, PGE2, NO	Cartilage	Downregulating of type II collagen and aggrecan gene expression, enhancing catabolism, reducing cartilage extracellular matrix (ECM) production in chondrocytes	[152, 153]
	P38	IL-1β, IL-6, MMP-1, MMP-13, ADAMTS-4, PGE2, NO	Cartilage	Enhancing catabolism, reducing cartilage extracellular matrix (ECM) production in chondrocytes	[152, 153]
	JNK	IL-1β, IL-6, MMP-1, MMP-13, ADAMTS-4, ADAMTS-5, VEGF	Cartilage	Enhancing catabolism, reducing cartilage extracellular matrix (ECM) production in chondrocytes	[152, 153]
	AMPKα	IL-1β, TNF-α, MMP-3, MMP-13	Cartilage	Enhancing cartilage catabolism and promoting chondrocyte apoptosis	[24, 154, 155]
c-Fos/AP-1	c-Fos/AP-1	IL-1β, IL-6,	Cartilage Osteophyte	Enhancing cartilage destruction and osteophyte formation	[156]
Focal adhesion pathway	FAK	IL-1β, IL-6, IL-8, TNF-α, COX-2	Cartilage subchondral bone	Enhancing subchondral bone deterioration and cartilage degeneration	[157–159]
	Hic-5	IL-1β, TNF-α, MMP-13, ADAMTS-5	Cartilage	Enhancing cartilage catabolism	[160, 161]
	Integrinα5β1	IL-1β, TNF-α, MMP-1, MMP-2, MMP-3, MMP-10, MMP-13, PGE2, NO, ADAMTS-5	Cartilage	Decreased proliferation of cartilage producing cells, chondrodysplasia, disorganized articular cartilage, and growth plate abnormalities	[162, 163]
FoxO3A	FoxO3A	IL-1β, TNF-α, MMP-3, MMP-13, iNOS	Cartilage	Inhibiting the progression of cartilage damage	[164, 165]
FGF pathway	FGF2	IL-1β, IL-6, IL-8, TNF-α, MMP-9, MMP-13, MCP-1, CCL2, ADAMTS-5	Cartilage	Promoting matrix degradation, anti-anabolism, and catabolism, enhancing cartilage destruction	[166–169]
HIFs pathway	HIF-1α	IL-1β, IL-6, TNF-α, MMP-13, ADAMTS-5, iNOS, VEGF, PGE2, NOS	Cartilage Synovium	Potentiating the synthesis of ECM, preventing cartilage degeneration	[170, 171]
	HIF-2α	IL-1β, IL-6 MMP-1, MMP-3, MMP-9, MMP-12, MMP-13, ADAMTS-4, NOS2, COX2	Cartilage	Enhancing cartilage destruction	[171–173]
Hippo-YAP	YAP	IL-1β, TNF-α, MMP-3, MMP-13, ADAMTS-4, ADAMTS-5	Cartilage	Attenuating cartilage destruction	[174, 175]
JAK/STAT pathway	JAK/STAT	IL-1β, TNF-α, IL-4, IL-6, IL-10, IL-12, IL-13, IL-23, MMP-1, MMP-3, MMP13, ADAMTS-4, ADAMTS-5	Cartilage	Promoting ECM degradation and reducing type II collagen expression in chondrocytes	[176, 177]
mTOR pathway	mTOR	IL-1β, IL-6, TNF-α, MMP-3, MMP-9, MMP-13, COX-2, iNOs, ADAMTS-5	Cartilage subchondral bone Synovium	Enhancing osteophyte formation, subchondral sclerosis, osteophyte formation and synovial inflammation Enhancing autophagy and inhibiting the apoptosis of chondrocytes	[178, 179]
NF-κB pathway	RelA/p65	IL-1β, IL-6, TNF-α, MMP-1, ADAMTS-5, NOS2, COX2	Cartilage synovium	Enhancing cartilage degradation, aggrecan loss, and cartilage erosion	[180–183]
	NF-кB1/ p105p50	IL-1β, IL-6, TNF-α	Cartilage	Enhancing cartilage degradation, aggrecan loss, and cartilage erosion	[184, 185]
	IκB	IL-1β, IL-6, MMP-13	Cartilage Synovium	Enhancing cartilage catabolism	[181, 186, 187]
	IKKα/β	IL-1, NOS2, COX2, MMP-13, ADAMTS-5	Cartilage	Inhibiting IKK activity significantly, preventing IKB phosphorylation, enhancing chondrocyte catabolism and cartilage degeneration	[181, 186, 188]
	NFκB/PI3K/AKT	IL-1β, IL-6, TNF-α, MMP-1, MMP-13, ADAMTS-5, NO, PGE2	Cartilage	Enhancing cartilage catabolism and degeneration	[152, 178, 189]
	NF-κB/ELF3	IL-1β, IL-6, TNF-α, LPS, COX2, iNOS, MMP-13	Cartilage	Inducing the expression of matrix-degrading enzymes, regulating chondrocyte catabolism, enhancing cartilage degradation	[190–192]
	NF-κB/Notch1	IL-1β, IL-6, IL- 8, TNF- α	Cartilage	Impaired synthesis of cartilage-specific extracellular matrix, enhancing cartilage catabolism	[152, 193, 194]
PPAR	PPARα/γ	IL-1β, TNF-α, MMP-1, MMP-3, MMP-9, MMP-13, AGEs, NO, PGE2	Cartilage Synovium	Inhibiting catabolism and inflammatory-related factors, attenuating cartilage damage	[195–197]
PGC-1α	PGC-1α	IL-1β, IL-8, TNF-α, MMP-13, COX-2	Cartilage	Regulating the metabolic abnormalities, inhibiting chondrocyte apoptosis	[164, 198]
SIRT	SIRT1	IL-1β, TNF- α, MMP-13, ADAMTS-5	Cartilage	Inhibiting catabolism	[199, 200]
	SIRT3	IL-1β, TNF- α MMP-3, MMP-13, COX2, iNOS	Cartilage	Inhibiting inflammation and apoptosis, preventing cartilage damage	[201, 202]
	SIRT6	IL-1β, IL-4, IL-8, TNF-α, MMP-2, MMP-9, COX2, PAI-1	Cartilage Synovium	Inhibiting ECM degradation, preventing cartilage damage	[203–205]
TAK1	TAK1	IL-1β, IL-6, TNF-α, MMP-1 MMP-3, MMP-13, VEGF, COX-2, PGE2	Cartilage Synovium	Enhancing cartilage destruction, synovial inflammation	[206–208]
TGFβ /BMP	BMP2	IL-1, TNF- α, MMP-13	Cartilage	Inhibiting cartilage degeneration	[209, 210]
	BMP7	IL-1, IL-6, IL-10, MMP-1, MMP-13	Cartilage	Inhibiting cartilage degeneration	[211–213]
	TGFβs	IL-1, IL-6, TNF- α MMP-3, MMP-9, MMP-13, ADAMTS-5	Cartilage	Counteracting the suppression of proteoglycan synthesis	[211, 214, 215]
TLRs	TLRs	IL-1β, TNF-α, IL-6,IL-8, IL-12, IL-17, CCL5, NO	Cartilage	Increasing the shift from anabolism to a catabolism, enhancing cartilage degradation	[152, 216]
Wnt pathway	β-catenin	IL-1β, IL-6, TNF-α MMP-1, MMP-3, MMP-13, ADAMTS-5	Cartilage	Enhancing cartilage degradation	[217]
	Wnt-3A	IL-1β, MMP-1, MMP-3, MMP-13	Cartilage	Attenuating catabolism	[168, 217]
	Wnt-5A	IL-1β, MMP-1, MMP-3, MMP-9, MMP-13	Cartilage	Anti-anabolic and enhancing catabolism, inhibiting type II collagen	[152, 217]
	Wnt-7A/β-catenin	IL-1β, MMP-1, MMP-3, MMP-13	Cartilage	Inhibiting type II collagen	[152, 168, 217]
	Wnt-7B/β-catenin	IL-1β, IL-6, TNF-α, MMP-1, MMP-3, MMP-13	Cartilage	Enhancing cartilage destruction	[217, 218]

Owing to the upregulation of IL-1β during the OA process, Zhao et al. tried to ablate IL-1β to ameliorate its progression [96]. After delivering a targeting CRISPR-Cas system with an adeno-associated virus (AAV), histology and μCT analyses were performed. The study demonstrated that CRISPR-mediated destruction of IL-1β significantly remitted the symptoms of posttraumatic osteoarthritis (PTOA). The same targets and similar editing strategies were confirmed by Karlsen et al. [220]. Meanwhile, Dooley et al. identified and targeted the functional structural domain of IL-16 by using the CRISPR-Cas system, and RNP complexes containing recombinant Cas9 coupled to guide RNA were delivered to cells via electroporation [221]. This study demonstrates the regulatory role of the CRISPR-Cas system in targeting inflammatory factors for chondrogenic differentiation. To address the problem of impaired cell regenerative capacity due to the development of inflammatory conditions in the microenvironment of PTOA, Bonato et al. improved the concept of cartilage tissue engineering through the CRISPR-Cas system [222]. The study provided multivalent protection to inhibit signaling that activates proinflammatory and catabolism of NF-κB pathways by targeted knockdown of TGF-β-activated kinase 1 (TAK1) in cells by CRISPR-Cas9. TAK1-konckout chondrocytes could efficiently integrate into natural cartilage even under proinflammatory conditions. Besides, results demonstrated that TAK1-knockout chondrocytes secrete less cytokines, which in turn reduces the recruitment of proinflammatory M1 macrophages. This type of targeted CRISPR-Cas-engineered chondrocytes (cartilage tissues) for inflammatory conditions represents a new option for OA treatment. Notably, owing to the persistence of inflammatory factors in the OA synovium, inflammation-related changes in the microenvironment also affect a variety of autologous cellular strategies by promoting fibrocartilage deposition [223]. In addition to the engineering of autologous chondrocytes by altering inflammation-related genes, another promising approach is to combine mesenchymal stem cells (MSCs) with the CRISPR-Cas system to attenuate inflammatory signals that promote ECM degradation, especially targeting IL-1Ra [223–225]. Another common CRISPR-Cas9-edited inflammation-associated stem cell is the induced pluripotent stem cell (iPSC) to improve immunomodulation of arthritis. CRISPR-Cas9-edited iPSCs targeted loss of IL-1R, thereby preventing IL-1-induced inflammatory responses and subsequent tissue degradation [226]. Recently, an engineered iPSC with a dynamic negative feedback loop was constructed using CRISPR-Cas9 technology and mouse iPSCs by Brunger et al. [227]. By adding IL-1Ra or soluble TNFR1 (Tnfrsf1a) genes downstream of the Ccl2 promoter, iPSCs can synthesize anticytokines under IL-1 or TNF-α stimulation in a self-regulatory fashion and effectively inhibit inflammation in a self-regulatory manner. The model has already been used for inflammation in animal models of rheumatoid arthritis (RA) [228]. Considering that OA and RA are also osteoarticular inflammatory diseases involving the synovium and joints, this scheme may provide a new direction for gene inflammation therapy of OA. With the growing understanding of the mechanisms of inflammation and corresponding immune regulation, CRISPR-Cas9-mediated Treg therapies have improved arthritis treatment, although the transmission, lifespan, and plasticity of these cells in vivo are unknown [229].

In summary, the use of CRISPR-Cas9 technology to (1) directly knock down overexpressed inflammation-related genes in existing cells, (2) engineer delivered chondrocytes by inflammation-related gene edition, (3) perform gene edition of undifferentiated stem cells to make them antiinflammatory to cope with the postdifferentiation inflammatory milieu, and (4) edit various genes of effector cells that perform immunomodulatory functions in inflammatory environments are the directions of OA gene therapy for inflammation.

## Application of the CRISPR-Cas system for cartilage repair in the process of OA

Cartilage defects are the most critical feature of OA progression [230]. Owing to the complexity of cellular components in the microenvironment in which articular cartilage resides (e.g., chondrocytes, immune cells, endothelial cells, synoviocytes, adipocytes, mesenchymal stem cells, etc.), the repair of cartilage defects is comodulated by the intercommunication of multiple cytokines [231]. In particular, dysfunctional chondrocytes that have undergone a series of stimuli such as senescence and inflammation release excessive amounts of protease matrix-degrading enzymes (typically composed of MMPs and ADAMTSs) in response to persistent stimuli in the OA environment, which induces proinflammatory factors to be released from neighboring cells and further enhances the activity of these enzymes, ultimately contributing to the persistence of low-grade inflammation and local tissue damage [232]. On the basis of the existence of the vicious circle in the microenvironment described above, cartilage defects become increasingly severe, and the repair of cartilage tissue will be severely impeded. More importantly, although articular cartilage is durable, it lacks blood vessels, resulting in poor regeneration and limited intrinsic healing [233]. Existing cartilage repair strategies include microfractures, autologous chondrocyte cell transplantation, biomaterial-based scaffolding techniques for cartilage repair, and various tissue engineering techniques. However, there has not yet been a technique that meets all the requirements for successful cartilage healing, i.e., embodying appropriate bioactivity, structure-function relationships, and ECM organization relationships [231]. Thus, combining gene therapy, cell/tissue engineering, and biomaterials as crosslinking projects may provide a potential direction.

Among the various types of cartilage repair concepts that have emerged in recent years, the utilization of MSCs is currently one of the most promising ideas [234]. As research has clarified that chondrocytes are one of the many types of cells that differentiate from MSCs [235], several current studies are exploring how to appropriately engineer MSCs to adapt them to the needs of cartilage repair. One of the prevailing ideas in this regard is to reprogram cells to give them special abilities [234]. CRISPR-Cas-based introduction of exogenous genes and regulation of gene expression levels and engineering of MSCs for regenerative medicine has grown significantly. The core idea of engineering MSCs using CRISPR-Cas is to replace the diseased cells and integrate them into the target tissue to achieve a therapeutic effect while avoiding an inflammatory response [236]. MSCs have the differentiation potential to receive physical, chemical, and biological stimuli for lineage transformation and ultimately directed differentiation, and the genes, transcription factors, microRNAs, and signaling pathways involved in the whole process will be activated or inhibited, which facilitates the application of the CRISPR-Cas system [237–239]. For example, RNA-guided nucleases (RGNs) in combination with the CRISPR system can be targeted to increase the expression of antiinflammatory factor genes in order to delay the progression of arthritis [240]. Aggrecan, type II collagen, and SOX9 are considered to be the major transcription factors involved in the differentiation of MSCs into chondrocytes [232, 237, 241], which can be targeted to enhance the potential of MSCs for cartilage repair. The use of CRISPR-Cas9 technology can also delay telomere shortening and reduce histone deacetylation as well as DNA methylation [242–244]. Owing to its capability for multigene editing, it can be used to promote chemokine receptor expression to increase MSC homing and adhesion to target tissues while having an anti-aging effect [242]. These studies show great promise for genome editing by the CRISPR-Cas system in engineering stem cells for cartilage repair therapeutic applications, but several ethical issues regarding the possible ethical implications of cytogenetic manipulation still need to be resolved before its use in clinical practice.

In addition to improving aging and suppressing local inflammation to slow the progression of osteoarthritis and enhance cartilage repair, another important idea is to maintain chondrocyte homeostasis, enhance differentiation of chondrocytes, and reduce apoptosis of extant chondrocytes and breakdown of differentiated cartilage components. Nowadays, various types of RNA have been used as potential therapeutic targets. Based on microRNA 140 (miR-140) known as a chondrocyte-specific endogenous gene regulator associated with osteoarthritis, Chaudhry et al. highly efficiently edited products targeting miR-140 gene editing were obtained using two sgRNAs in combination with dual RNP-mediated CRISPR-Cas9 transfection [245]. The results indicate that this targeted removal of miR-140 can significantly improve the expression levels of a variety of genes in chondrocytes, especially for genes that require high removal levels to observe significant expression differences. Nguyen et al. focused on LncRNA DANCR, which induces differentiation of human synovial-derived stem cells into cartilage. By leveraging the superior ability to edit targets and upregulate expression of dCas9 compared with conventional Cas9, they successfully induced the activation of DANCR in adipose-derived stem cells after screening by packaging dCas9 and the corresponding gRNA against DANCR in viruses and delivering them, which provides a new idea for the repair of cartilage defects [112]. Additionally, since MMP13 was identified as a major factor affecting type II collagen content, numerous studies have focused on how targeted knockdown of the MMP13 gene can ameliorate type II collagen loss. Sedil et al. used a CRISPR/Cas9-mediated gene editing strategy to reconstruct human chondrocytes lines and achieved a stable reduction of MMP13 expression in chondrocytes. The reduction of total MMP13 secretion by CRISPR/Cas9 indirectly reduced the degradation of ECM and increased the concentration of type II collagen [246]. Meanwhile, to solve the decomposition problem of CRISPR-Cas therapeutic molecules during delivery and to enhance the therapeutic effect, Liang et al. used cartilage-targeted exosomes for direct delivery to knock down the MMP13 gene and achieved a more significant therapeutic effect [110]. The publication of this study suggests that CRISPR-Cas therapy has stepped into new territory. The classical targets also include aggrecan and type II collagen. The study confirmed that the use of dCas9 to induce dual overexpression of the two can effectively achieve the deposition of sGAG and type II collagen, provide better support for the ECM, control chondrocyte growth and differentiation, and better regulate the cell phenotype [247, 248]. And essentially, the original purpose of CRISPR-Cas was to modify mutated genes to fundamentally alter the various types of hereditary diseases and cancers that result from genetic mutations. The use of gene mutation therapy based on this idea to achieve the realization of gene upregulation or the correction of mutations during cartilage repair is a new idea. Nonaka et al. used CRISPR to repair a functional single-base mutation in transient receptor potential vanilloid 4 (TRPV4). The mutation leads to an increase in calcium ions and ultimately to ectopic dysplasia. The experimental results demonstrated that the mutant group showed significantly accelerated chondrogenic differentiation and SOX9 mRNA expression [249].

Recently, it has been increasingly recognized that OA is also a mitochondrial disease [250]. Mitochondria from diseased chondrocytes show a significant increase in mass, reduced capacity of antioxidant enzymes, decreased activity of respiratory complexes, and overproduction of reactive oxygen species (ROS) and reactive nitrogen species (RNS) compared with healthy cells [251–253]. Current studies demonstrate that the changes are highly correlated to mutations in mitochondria DNA (mtDNA) [254]. Once the mutation occurs, it will easily generate proteases that lead to mitochondrial oxidation and phosphorylation, resulting in mitochondrial dysfunction and damage [255]. In addition, mtDNA is susceptible to exogenous stimulation and has a high probability of mutation [250]. Although mitochondria have the function to repair their own mtDNA through a series of ways such as double-bond break repair and base excision repair, it is not realistic to maintain mitochondrial homeostasis under extreme environments (e.g., OA) through this fragile self-repair ability [256, 257]. Once such damage reaches a threshold, mtDNA damage will lead to mitochondrial pathological phenotypic changes and lasting impairment of physiological functions, causing disruption of metabolism within chondrocytes [258, 259]. Gene editing targeting mitochondria to treat OA has better prospects, such as targeting mitochondria with peptide nucleic acids complementary to mtDNA templates to inhibit replication of mutant sequences [260–262], using mitochondria-targeted restriction endonucleases to alter DNA specificity and reduce genomic mutations [263], using zinc finger enzymes to recognize and eliminate the effects of mutations [264, 265], etc. The emergence of the CRISPR-Cas system offers more potential for mtDNA editing, and repair offers even more promising possibilities. There have been studies using CRISPR-Cas9 to target COX1 and COX3 in mtDNA to achieve mitochondrial membrane potential disruption and cell growth inhibition [266]. However, owing to the natural barrier effect of the mitochondrial bilayer membrane structure on sgRNA and the off-target risk of CRISPR itself, its further application needs more exploration [250, 267]. Although the therapeutic application of mitochondrial genome editing in OA is still relatively unstudied, it is possible to target mutant mitochondrial genes leading to OA-associated oxidation by correcting altered phenotypes through CRISPR or by integrating suitable genes, even involving differentiation or regeneration gene sequences [250].

## Prospects and conclusions

**Fig. 3:**
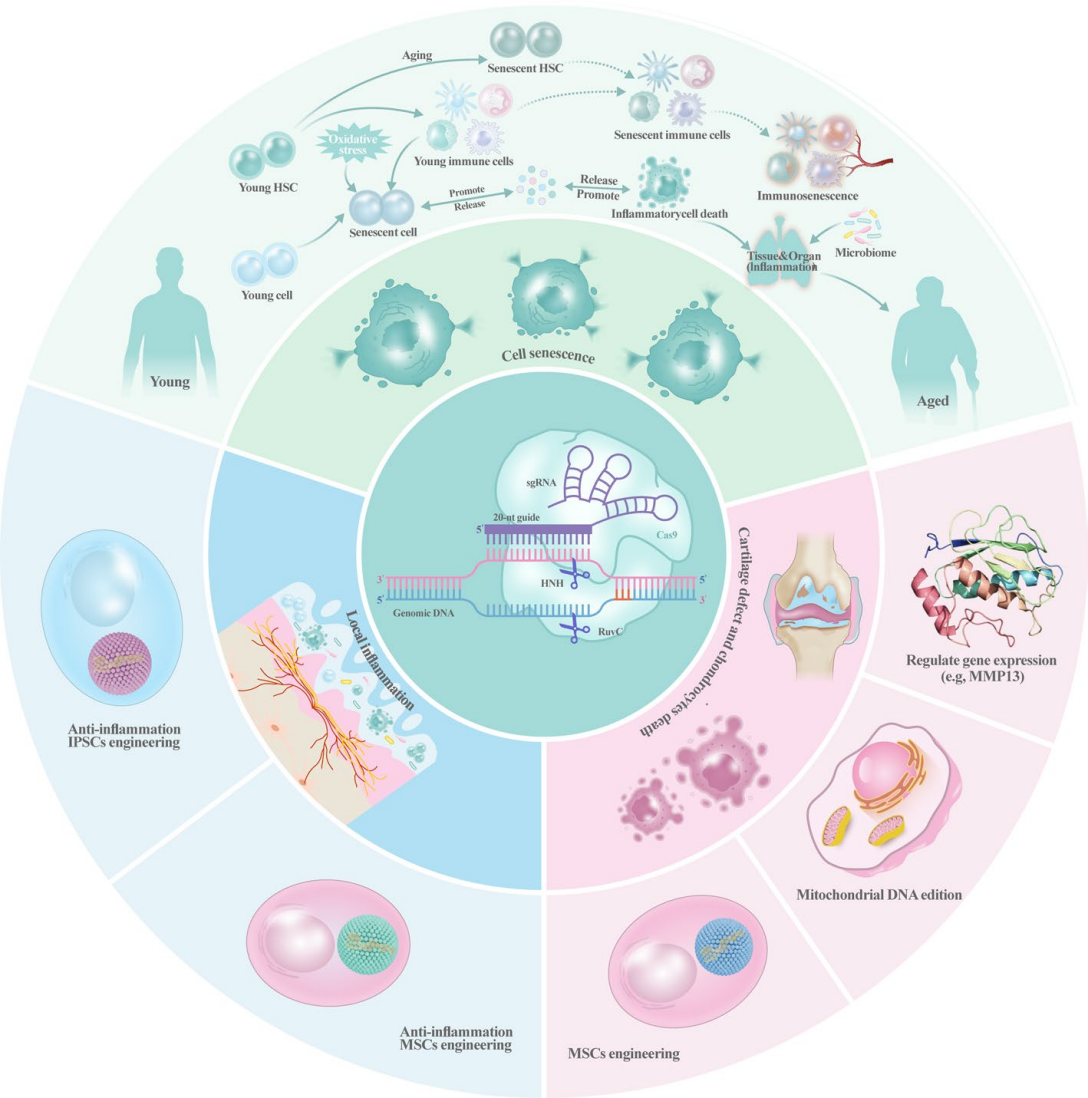
An overview of strategies for OA treatment based on the CRISPR-Cas system. The CRISPR-Cas system treats OA through three main pathways: inhibiting release of senescence-associated factors and regulating senescence-associated immune processes, implanting gene-edited stem cells and chondrocytes in vivo to enhance their function, or modulating the inflammatory pathways involved in the process of OA

Since the emergence of CRISPR-Cas technology, it has played an important role in many fields such as the life sciences, medicine, and bioengineering, boasting unique advantages such as high precision, efficiency, simplicity, and broad applicability from therapeutic interventions to agricultural enhancements. However, the following challenges still need to be solved: (1) off-target effects of CRISPR and subsequent safety issues, (2) crosstalk caused by the complex gene regulation of OA and the still-unspecified multiple potential target genes, and (3) inefficiency due to gene editing of individual chondrocytes. Orthopedic researchers are working hard to apply this cross-generational tool to their relevant fields. Although its large-scale applications are currently limited to tumors, or congenital or genetic diseases, some researchers are still hoping to broaden the boundaries of its use to address the increasing severity of OA and its underlying cartilage repair problems, with a view to conquering the “cancer that never dies.” Given that OA occurs and progresses because of cellular senescence and apoptosis under natural or stressful conditions, as well as inflammation, including trauma, this paper reviews the relevant mechanistic pathways and the current applications of CRISPR-Cas technology in reversing OA-associated cellular senescence, improving the inflammatory microenvironment, and thus promoting cartilage repair (Fig. 3). In general, the main methods of CRISPR-Cas technology for OA gene therapy are (1) in vivo injection of the CRISPR-Cas system to change the phenotype of existing cells or reduce the formation of related harmful metabolites, (2) in vitro gene editing of chondrocytes, synoviocytes, or various types of senescent cells, which are then reimplanted into organisms for therapeutic purposes, and (3) engineering of undifferentiated stem cells, such as MSCs, to endow them with the ability to repair the inflammatory microenvironment (Fig. 3) or differentiated stem cells, such as MSCs, to endow them with antiinflammatory, anti-aging, and rapid directional differentiation into chondrocytes, so that they can survive under the extreme environment of OA and rapidly differentiate into chondrocytes for repairing damaged cartilage, and (4) genetically editing the mitochondrial DNA of damaged chondrocytes to improve or even reverse the energetic homeostasis of the damaged cells, to maintain the cellular lifespan. Owing to ethical issues, fundamental embryo editing to create an “OA-free” population is unavailable. More random controlled trials (RCTs) and follow-up should be conducted to prove safety and efficacy, as well as alleviate concerns based on ethical issues. Currently, the application of CRISPR-Cas in the field of the musculoskeletal system is mostly focused on rheumatoid arthritis with synovial membrane damage and various types of bone tumors. Reasonable use of relevant vectors to knock down disease-causing genes or overexpress antagonist genes to achieve eradication at the transcriptional level and significantly improve the efficacy in inflammatory or immune diseases and obtain specific phenotypes by knocking down deserve further research effort. Although OA is affected by multiple factors, the relevant target factors are being gradually and one by one validated. Broadening the boundaries of OA gene therapy beyond these avenues holds broad prospects and great research value.

## Data Availability

Not acceptable.

## Abbreviations

OA: Osteoarthritis
CRISPR: Clustered regularly interspaced short palindromic repeats
Cas: CRISPR associated
crRNA: CRISPR RNA
RCTs: Random controlled trials
NSAIDs: Nonsteroidal antiinflammatory drugs
EVs: Extracellular vesicles
dsDNA: Double-strand DNA
gRNA: Guide RNA
PAM: Proto-spacer adjacent motif
tracrRNA: Trans-activating crRNA
REC: Recognition lobe
NUC: Nuclease lobe
CTD: C-terminal domain
sgRNA: Single guide RNA
DSB: Double-strand break
NHEJ: Nonhomologous end joining
HR: Homologous recombination
dCas9: Dead-Cas9
ssDNA: Single-strand DNA
ECM: Extracellular matrix
SASP: Senescence-associated secretory phenotype
FOXO: Forkhead box subgroup O
CDKN1B: Cyclin-dependent kinase inhibitor 1B
RBL2: RB transcriptional corepressor like 2
VEGF: Vascular endothelial growth factor
YAP: Yes-associated protein
hMSCs: Human mesenchymal stem cells
SAM: Synergistic activation mediator
CRISPRa: CRISPR-based activation
SOX5: SRY-Box transcription factor 5
HMGB2: High-mobility group box 2
ROS: Reactive oxygen species
Cx43: Connexin 43
GJIC: Gap junctional intercellular communication
IL-1β: Interleukin-1β
IL-1R: IL-1 receptor
TNF-α: Tumor necrosis factor-α
DMOADs: Disease-modifying OA drugs
AAV: Adeno-associated virus
PTOA: Posttraumatic osteoarthritis
TAK1: TGF-β-activated kinase 1
MSCs: Mesenchymal stem cells
iPSCs: Induced pluripotent stem cells
TNFR1: Tnfrsf1a
RA: Rheumatoid arthritis
RGN: RNA-guided nucleases
RNS: Reactive nitrogen species
mtDNA: Mitochondrial DNA
TPRV4: Transient receptor potential vanilloid 4
LNPs: Lipid nanoparticles

## References

[1] Yao Q, Wu X, Tao C, Gong W, Chen M, Qu M (2023). Osteoarthritis: pathogenic signaling pathways and therapeutic targets. Signal Transduct Target Ther.

[2] Chen D, Shen J, Zhao W, Wang T, Han L, Hamilton JL (2017). Osteoarthritis: toward a comprehensive understanding of pathological mechanism. Bone Res.

[3] Chen D (2022). Osteoarthritis: a complicated joint disease requiring extensive studies with multiple approaches. J Orthop Translat.

[4] Sun AR, Udduttula A, Li J, Liu Y, Ren PG, Zhang P (2021). Cartilage tissue engineering for obesity-induced osteoarthritis: physiology, challenges, and future prospects. J Orthop Transl.

[5] Li J, Zhang B, Liu WX, Lu K, Pan H, Wang T (2020). Metformin limits osteoarthritis development and progression through activation of AMPK signalling. Ann Rheum Dis.

[6] Li J, Wang Y, Chen D, Liu-Bryan R (2022). Oral administration of berberine limits post-traumatic osteoarthritis development and associated pain via AMP-activated protein kinase (AMPK) in mice. Osteoarthr Cartil.

[7] Evans CH, Ghivizzani SC, Robbins PD (2023). Osteoarthritis gene therapy in 2022. Curr Opin Rheumatol.

[8] Ruud J, JanDAV E, Wim G, LeoM S (2002). Identification of genes that are associated with DNA repeats in prokaryotes. Mol Microbiol.

[9] Marraffini LA (2015). CRISPR-Cas immunity in prokaryotes. Nature.

[10] Mojica FJM, Rodriguez-Valera F (2016). The discovery of CRISPR in archaea and bacteria. FEBS J.

[11] Wang SW, Gao C, Zheng YM, Yi L, Lu JC, Huang XY (2022). Current applications and future perspective of CRISPR/Cas9 gene editing in cancer. Mol Cancer.

[12] Jiang F, Doudna JA (2017). CRISPR-Cas9 structures and mechanisms. Annu Rev Biophys.

[13] Wiedenheft B, Sternberg SH, Doudna JA (2012). RNA-guided genetic silencing systems in bacteria and archaea. Nature.

[14] van der Oost J, Westra ER, Jackson RN, Wiedenheft B (2014). Unravelling the structural and mechanistic basis of CRISPR-Cas systems. Nat Rev Microbiol.

[15] Eş I, Gavahian M, Marti-Quijal FJ, Lorenzo JM, Khaneghah AM, Tsatsanis C (2019). The application of the CRISPR-Cas9 genome editing machinery in food and agricultural science: current status, future perspectives, and associated challenges. Biotechnol Adv.

[16] Li C, Du Y, Zhang T, Wang H, Hou Z, Zhang Y (2023). “Genetic scissors” CRISPR/Cas9 genome editing cutting-edge biocarrier technology for bone and cartilage repair. Bioact Mater.

[17] Tong L, Yu H, Huang X, Shen J, Xiao G, Chen L (2022). Current understanding of osteoarthritis pathogenesis and relevant new approaches. Bone Res.

[18] Loeser RF, Collins JA, Diekman BO (2016). Ageing and the pathogenesis of osteoarthritis. Nat Rev Rheumatol.

[19] Collins JA, Diekman BO, Loeser RF (2018). Targeting aging for disease modification in osteoarthritis. Curr Opin Rheumatol.

[20] Hwang HS, Kim HA (2015). Chondrocyte apoptosis in the pathogenesis of osteoarthritis. Int J Mol Sci.

[21] Castrogiovanni P, Ravalli S, Musumeci G (2020). Apoptosis and autophagy in the pathogenesis of osteoarthritis. J Invest Surg.

[22] Hu J, Zhou J, Wu J, Chen Q, Du W, Fu F (2020). Loganin ameliorates cartilage degeneration and osteoarthritis development in an osteoarthritis mouse model through inhibition of NF-κB activity and pyroptosis in chondrocytes. J Ethnopharmacol.

[23] Sun MM, Beier F (2014). Chondrocyte hypertrophy in skeletal development, growth, and disease. Birth Defects Res Pt C.

[24] Zheng L, Zhang Z, Sheng P, Mobasheri A (2021). The role of metabolism in chondrocyte dysfunction and the progression of osteoarthritis. Ageing Res Rev.

[25] Chen D, Kim DJ, Shen J, Zou Z, O’Keefe RJ (2020). Runx2 plays a central role in osteoarthritis development. J Orthop Transl.

[26] Funck-Brentano T, Cohen-Solal M (2015). Subchondral bone and osteoarthritis. Curr Opin Rheumatol.

[27] Hui W, Young DA, Rowan AD, Xu X, Cawston TE, Proctor CJ (2016). Oxidative changes and signalling pathways are pivotal in initiating age-related changes in articular cartilage. Ann Rheum Dis.

[28] Blom AB, Brockbank SM, Van Lent PL, Van Beuningen HM, Geurts J, Takahashi N (2009). Involvement of the Wnt signaling pathway in experimental and human osteoarthritis: prominent role of Wnt-induced signaling protein 1. Arthritis Rheum.

[29] Chen Y, Luo X, Kang R, Cui K, Ou J, Zhang X (2023). Current therapies for osteoarthritis and prospects of CRISPR-based genome, epigenome, and RNA editing in osteoarthritis treatment. J Genet Genomics.

[30] Silverwood V, Blagojevic-Bucknall M, Jinks C, Jordan JL, Protheroe J, Jordan KP (2015). Current evidence on risk factors for knee osteoarthritis in older adults: a systematic review and meta-analysis. Osteoarthr Cartil.

[31] Glyn-Jones S, Palmer AJR, Agricola R, Price AJ, Vincent TL, Weinans H (2015). Osteoarthritis. Lancet.

[32] Peat G, Thomas MJ (2021). Osteoarthritis year in review 2020: epidemiology and therapy. Osteoarthr Cartil.

[33] Zhang J, Liu GH, Qu J, Song M (2020). Treating osteoarthritis via gene therapy with rejuvenation factors. Gene Ther.

[34] Hunter DJ, Bierma-Zeinstra S (2019). Osteoarthritis. Lancet.

[35] Kim JH, Kim HJ, Lee DH (2017). Survival of opening versus closing wedge high tibial osteotomy: a meta-analysis. Sci Rep.

[36] van der Woude JAD, Wiegant K, van Heerwaarden RJ, Spruijt S, van Roermund PM, Custers RJH (2017). Knee joint distraction compared with high tibial osteotomy: a randomized controlled trial. Knee Surg Sports Traumatol Arthrosc.

[37] Yoon KH, Park JY, Lee JY, Lee E, Lee J, Kim SG (2020). Costal chondrocyte-derived pellet-type autologous chondrocyte implantation for treatment of articular cartilage defect. Am J Sports Med.

[38] Yoon KH, Yoo JD, Choi CH, Lee J, Lee JY, Kim SG (2021). Costal chondrocyte-derived pellet-type autologous chondrocyte implantation versus microfracture for repair of articular cartilage defects: a prospective randomized trial. Cartilage.

[39] Yoon KH, Lee J, Park JY (2024). Costal chondrocyte-derived pellet-type autologous chondrocyte implantation versus microfracture for the treatment of articular cartilage defects: a 5-year follow-up of a prospective randomized trial. Am J Sports Med.

[40] Arthur A, Zannettino A, Gronthos S (2009). The therapeutic applications of multipotential mesenchymal/stromal stem cells in skeletal tissue repair. J Cell Physiol.

[41] Caron MMJ, Emans PJ, Coolsen MME, Voss L, Surtel DAM, Cremers A (2012). Redifferentiation of dedifferentiated human articular chondrocytes: comparison of 2D and 3D cultures. Osteoarthr Cartil.

[42] Zhang S, Teo KYW, Chuah SJ, Lai RC, Lim SK, Toh WS (2019). MSC exosomes alleviate temporomandibular joint osteoarthritis by attenuating inflammation and restoring matrix homeostasis. Biomaterials.

[43] Cosenza S, Ruiz M, Toupet K, Jorgensen C, Noël D (2017). Mesenchymal stem cells derived exosomes and microparticles protect cartilage and bone from degradation in osteoarthritis. Sci Rep.

[44] He L, He T, Xing J, Zhou Q, Fan L, Liu C (2020). Bone marrow mesenchymal stem cell-derived exosomes protect cartilage damage and relieve knee osteoarthritis pain in a rat model of osteoarthritis. Stem Cell Res Ther.

[45] Tofiño-Vian M, Guillén MI, Pérez Del Caz MD, Silvestre A, Alcaraz MJ (2018). Microvesicles from human adipose tissue-derived mesenchymal stem cells as a new protective strategy in osteoarthritic chondrocytes. Cell Physiol Biochem.

[46] Schmidt MB, Chen EH, Lynch SE (2006). A review of the effects of insulin-like growth factor and platelet derived growth factor on in vivo cartilage healing and repair. Osteoarthr Cartil.

[47] Morscheid YP, Venkatesan JK, Schmitt G, Orth P, Zurakowski D, Speicher-Mentges S (2021). rAAV-mediated human FGF-2 gene therapy enhances osteochondral repair in a clinically relevant large animal model over time in vivo. Am J Sports Med.

[48] Ashraf S, Kim BJ, Park S, Park H, Lee SH (2019). RHEB gene therapy maintains the chondrogenic characteristics and protects cartilage tissue from degenerative damage during experimental murine osteoarthritis. Osteoarthr Cartil.

[49] Zhang DW, Yang QS, Zhu JY, Cao XR, Li LW, Zhu QS (2007). Amelioration of osteoarthritis by intra-articular hyaluronan synthase 2 gene therapy. Med Hypotheses.

[50] Chen X, Shi Y, Xue P, Ma X, Li J, Zhang J (2020). Mesenchymal stem cell-derived exosomal microRNA-136-5p inhibits chondrocyte degeneration in traumatic osteoarthritis by targeting ELF3. Arthritis Res Ther.

[51] Cao Y, Tang S, Nie X, Zhou Z, Ruan G, Han W (2021). Decreased miR-214-3p activates NF-κB pathway and aggravates osteoarthritis progression. EBioMedicine.

[52] Qin H, Wang C, He Y, Lu A, Li T, Zhang B (2023). Silencing miR-146a-5p protects against injury-induced osteoarthritis in mice. Biomolecules.

[53] Bedingfield SK, Colazo JM, Yu F, Liu DD, Jackson MA, Himmel LE (2021). Amelioration of post-traumatic osteoarthritis via nanoparticle depots delivering small interfering RNA to damaged cartilage. Nat Biomed Eng.

[54] Winkle M, El-Daly SM, Fabbri M, Calin GA (2021). Noncoding RNA therapeutics—challenges and potential solutions. Nat Rev Drug Discov.

[55] Makarova KS, Wolf YI, Iranzo J, Shmakov SA, Alkhnbashi OS, Brouns SJJ (2020). Evolutionary classification of CRISPR-Cas systems: a burst of class 2 and derived variants. Nat Rev Microbiol.

[56] Shmakov S, Smargon A, Scott D, Cox D, Pyzocha N, Yan W (2017). Diversity and evolution of class 2 CRISPR–Cas systems. Nat Rev Microbiol.

[57] Jolany Vangah S, Katalani C, Boone HA, Hajizade A, Sijercic A, Ahmadian G (2020). CRISPR-based diagnosis of infectious and noninfectious diseases. Biol Proced Online.

[58] Garneau JE, Dupuis MÈ, Villion M, Romero DA, Barrangou R, Boyaval P (2010). The CRISPR/Cas bacterial immune system cleaves bacteriophage and plasmid DNA. Nature.

[59] Jinek M, Chylinski K, Fonfara I, Hauer M, Doudna JA, Charpentier E (2012). A programmable Dual-RNA–guided DNA endonuclease in adaptive bacterial immunity. Science.

[60] Yeh CD, Richardson CD, Corn JE (2019). Advances in genome editing through control of DNA repair pathways. Nat Cell Biol.

[61] Bian J, Cai F, Chen H, Tang Z, Xi K, Tang J (2021). Modulation of local overactive inflammation via injectable hydrogel microspheres. Nano Lett.

[62] Cong L, Ran FA, Cox D, Lin S, Barretto R, Habib N (2013). Multiplex genome engineering using CRISPR/Cas systems. Science.

[63] Cho SW, Kim S, Kim JM, Kim JS (2013). Targeted genome engineering in human cells with the Cas9 RNA-guided endonuclease. Nat Biotechnol.

[64] Mali P, Yang L, Esvelt KM, Aach J, Guell M, DiCarlo JE (2013). RNA-guided human genome engineering via Cas9. Science.

[65] Wang M, Chen M, Wu X, Huang X, Yu B (2023). CRISPR applications in cancer diagnosis and treatment. Cell Mol Biol Lett.

[66] Wang JY, Doudna JA (2023). CRISPR technology: a decade of genome editing is only the beginning. Science.

[67] De Bragança S, Dillingham MS, Moreno-Herrero F (2023). Recent insights into eukaryotic double-strand DNA break repair unveiled by single-molecule methods. Trends Genet.

[68] Fu YW, Dai XY, Wang WT, Yang ZX, Zhao JJ, Zhang JP (2021). Dynamics and competition of CRISPR–Cas9 ribonucleoproteins and AAV donor-mediated NHEJ, MMEJ and HDR editing. Nucleic Acids Res.

[69] Román-Rodríguez FJ, Ugalde L, Álvarez L, Díez B, Ramírez MJ, Risueño C (2019). NHEJ-mediated repair of CRISPR-Cas9-induced DNA breaks efficiently corrects mutations in HSPCs from patients with fanconi anemia. Cell Stem Cell.

[70] Branzei D, Foiani M (2008). Regulation of DNA repair throughout the cell cycle. Nat Rev Mol Cell Biol.

[71] Lin S, Staahl BT, Alla RK, Doudna JA (2014). Enhanced homology-directed human genome engineering by controlled timing of CRISPR/Cas9 delivery. Elife.

[72] Musunuru K (2017). The hope and hype of CRISPR-Cas9 genome editing: a review. JAMA Cardiol.

[73] Qi LS, Larson MH, Gilbert LA, Doudna JA, Weissman JS, Arkin AP (2013). Repurposing CRISPR as an RNA-guided platform for sequence-specific control of gene expression. Cell.

[74] Dominguez AA, Lim WA, Qi LS (2016). Beyond editing: repurposing CRISPR–Cas9 for precision genome regulation and interrogation. Nat Rev Mol Cell Biol.

[75] Yan WX, Hunnewell P, Alfonse LE, Carte JM, Keston-Smith E, Sothiselvam S (2019). Functionally diverse type V CRISPR-Cas systems. Science.

[76] Fonfara I, Richter H, Bratovič M, Le Rhun A, Charpentier E (2016). The CRISPR-associated DNA-cleaving enzyme Cpf1 also processes precursor CRISPR RNA. Nature.

[77] Chen JS, Ma E, Harrington LB, Da Costa M, Tian X, Palefsky JM (2018). CRISPR-Cas12a target binding unleashes indiscriminate single-stranded DNase activity. Science.

[78] Samai P, Pyenson N, Jiang W, Goldberg GW, Hatoum-Aslan A, Marraffini LA (2015). Co-transcriptional DNA and RNA cleavage during type III CRISPR-Cas immunity. Cell.

[79] Richardson CD, Ray GJ, DeWitt MA, Curie GL, Corn JE (2016). Enhancing homology-directed genome editing by catalytically active and inactive CRISPR-Cas9 using asymmetric donor DNA. Nat Biotechnol.

[80] Wu S, Tian P, Tan T (2022). CRISPR-Cas13 technology portfolio and alliance with other genetic tools. Biotechnol Adv.

[81] Abudayyeh OO, Gootenberg JS, Essletzbichler P, Han S, Joung J, Belanto JJ (2017). RNA targeting with CRISPR-Cas13. Nature.

[82] O'Connell MR (2019). Molecular mechanisms of RNA targeting by Cas13-containing Type VI CRISPR-Cas systems. J Mol Biol..

[83] East-Seletsky A, O’Connell MR, Knight SC, Burstein D, Cate JHD, Tjian R (2016). Two distinct RNase activities of CRISPR-C2c2 enable guide-RNA processing and RNA detection. Nature.

[84] Smargon AA, Shi YJ, Yeo GW (2020). RNA-targeting CRISPR systems from metagenomic discovery to transcriptomic engineering. Nat Cell Biol.

[85] Manghwar H, Lindsey K, Zhang X, Jin S (2019). CRISPR/Cas system: recent advances and future prospects for genome editing. Trends Plant Sci.

[86] Eş I, Gavahian M, Marti-Quijal FJ, Lorenzo JM, Mousavi Khaneghah A, Tsatsanis C (2019). The application of the CRISPR-Cas9 genome editing machinery in food and agricultural science: current status, future perspectives, and associated challenges. Biotechnol Adv.

[87] Huang X, Li A, Xu P, Yu Y, Li S, Hu L (2023). Current and prospective strategies for advancing the targeted delivery of CRISPR/Cas system via extracellular vesicles. J Nanobiotechnol.

[88] Lin J, Jia S, Jiao Z, Chen J, Li W, Cao F (2023). Global research trends in CRISPR-related technologies associated with extracellular vesicles from 2015 to 2022: a bibliometric, dynamic, and visualized study. Cell Mol Biol Lett.

[89] Wang HX, Li M, Lee CM, Chakraborty S, Kim HW, Bao G (2017). CRISPR/Cas9-based genome editing for disease modeling and therapy: challenges and opportunities for nonviral delivery. Chem Rev.

[90] Khoshandam M, Soltaninejad H, Mousazadeh M, Hamidieh AA, Hosseinkhani S (2024). Clinical applications of the CRISPR/Cas9 genome-editing system: delivery options and challenges in precision medicine. Genes Dis.

[91] Du Y, Liu Y, Hu J, Peng X, Liu Z (2023). CRISPR/Cas9 systems: delivery technologies and biomedical applications. Asian J Pharm Sci.

[92] Lee CS, Bishop ES, Zhang R, Yu X, Farina EM, Yan S (2017). Adenovirus-mediated gene delivery: potential applications for gene and cell-based therapies in the new era of personalized medicine. Genes Dis.

[93] Wang D, Tai PWL, Gao G (2019). Adeno-associated virus vector as a platform for gene therapy delivery. Nat Rev Drug Discov.

[94] Sun JY, Anand-Jawa V, Chatterjee S, Wong KK (2003). Immune responses to adeno-associated virus and its recombinant vectors. Gene Ther.

[95] Vakulskas CA, Behlke MA (2019). Evaluation and reduction of CRISPR Off-target cleavage events. Nucleic Acid Ther.

[96] Zhao L, Huang J, Fan Y, Li J, You T, He S (2019). Exploration of CRISPR/Cas9-based gene editing as therapy for osteoarthritis. Ann Rheum Dis.

[97] Kantor B, Bailey RM, Wimberly K, Kalburgi SN, Gray SJ (2014). Methods for gene transfer to the central nervous system. Adv Genet.

[98] Wanisch K, Yáñez-Muñoz RJ (2009). Integration-deficient lentiviral vectors: a slow coming of age. Mol Ther.

[99] Ling S, Yang S, Hu X, Yin D, Dai Y, Qian X (2021). Lentiviral delivery of co-packaged Cas9 mRNA and a Vegfa-targeting guide RNA prevents wet age-related macular degeneration in mice. Nat Biomed Eng.

[100] Waehler R, Russell SJ, Curiel DT (2007). Engineering targeted viral vectors for gene therapy. Nat Rev Genet.

[101] Li L, Hu S, Chen X (2018). Non-viral delivery systems for CRISPR/Cas9-based genome editing: challenges and opportunities. Biomaterials.

[102] Witzigmann D, Kulkarni JA, Leung J, Chen S, Cullis PR, van der Meel R (2020). Lipid nanoparticle technology for therapeutic gene regulation in the liver. Adv Drug Deliv Rev.

[103] Sercombe L, Veerati T, Moheimani F, Wu SY, Sood AK, Hua S (2015). Advances and challenges of liposome assisted drug delivery. Front Pharmacol.

[104] Han JP, Kim M, Choi BS, Lee JH, Lee GS, Jeong M (2022). In vivo delivery of CRISPR-Cas9 using lipid nanoparticles enables antithrombin gene editing for sustainable hemophilia A and B therapy. Sci Adv.

[105] Song R, Murphy M, Li C, Ting K, Soo C, Zheng Z (2018). Current development of biodegradable polymeric materials for biomedical applications. Drug Des Dev Ther.

[106] Yan M, Du J, Gu Z, Liang M, Hu Y, Zhang W (2010). A novel intracellular protein delivery platform based on single-protein nanocapsules. Nat Nanotechnol.

[107] Yang Z, Shi J, Xie J, Wang Y, Sun J, Liu T (2020). Large-scale generation of functional mRNA-encapsulating exosomes via cellular nanoporation. Nat Biomed Eng.

[108] Kamerkar S, LeBleu VS, Sugimoto H, Yang S, Ruivo CF, Melo SA (2017). Exosomes facilitate therapeutic targeting of oncogenic KRAS in pancreatic cancer. Nature.

[109] Vader P, Mol EA, Pasterkamp G, Schiffelers RM (2016). Extracellular vesicles for drug delivery. Adv Drug Deliv Rev.

[110] Liang Y, Xu X, Xu L, Iqbal Z, Ouyang K, Zhang H (2022). Chondrocyte-specific genomic editing enabled by hybrid exosomes for osteoarthritis treatment. Theranostics.

[111] Tsukamoto T, Sakai E, Iizuka S, Taracena-Gándara M, Sakurai F, Mizuguchi H (2018). Generation of the adenovirus vector-mediated CRISPR/Cpf1 system and the application for primary human hepatocytes prepared from humanized mice with chimeric liver. Biol Pharm Bull.

[112] Nguyen NTK, Chang YH, Truong VA, Hsu MN, Pham NN, Chang CW (2021). CRISPR activation of long non-coding RNA DANCR promotes bone regeneration. Biomaterials.

[113] Farbiak L, Cheng Q, Wei T, Álvarez-Benedicto E, Johnson LT, Lee S (2021). All-in-one dendrimer-based lipid nanoparticles enable precise HDR-mediated gene editing in vivo. Adv Mater.

[114] Liu C, Wan T, Wang H, Zhang S, Ping Y, Cheng Y (2019). A boronic acid-rich dendrimer with robust and unprecedented efficiency for cytosolic protein delivery and CRISPR-Cas9 gene editing. Sci Adv.

[115] Rosenblum D, Gutkin A, Kedmi R, Ramishetti S, Veiga N, Jacobi AM (2020). CRISPR-Cas9 genome editing using targeted lipid nanoparticles for cancer therapy. Sci Adv.

[116] Kenjo E, Hozumi H, Makita Y, Iwabuchi KA, Fujimoto N, Matsumoto S (2021). Low immunogenicity of LNP allows repeated administrations of CRISPR-Cas9 mRNA into skeletal muscle in mice. Nat Commun.

[117] Abbasi S, Uchida S, Toh K, Tockary TA, Dirisala A, Hayashi K (2021). Co-encapsulation of Cas9 mRNA and guide RNA in polyplex micelles enables genome editing in mouse brain. J Control Release.

[118] Bolduc JA, Collins JA, Loeser RF (2019). Reactive oxygen species, aging and articular cartilage homeostasis. Free Radic Biol Med.

[119] Roelofs AJ, De Bari C (2023). Osteoarthritis year in review 2023: Biology. Osteoarthr Cartil.

[120] Koike M, Nojiri H, Ozawa Y, Watanabe K, Muramatsu Y, Kaneko H (2015). Mechanical overloading causes mitochondrial superoxide and SOD2 imbalance in chondrocytes resulting in cartilage degeneration. Sci Rep.

[121] Rose J, Söder S, Skhirtladze C, Schmitz N, Gebhard PM, Sesselmann S (2012). DNA damage, discoordinated gene expression and cellular senescence in osteoarthritic chondrocytes. Osteoarthr Cartil.

[122] Harbo M, Bendix L, Bay-Jensen AC, Graakjaer J, Søe K, Andersen TL (2012). The distribution pattern of critically short telomeres in human osteoarthritic knees. Arthritis Res Ther.

[123] Loeser RF (2009). Aging and osteoarthritis: the role of chondrocyte senescence and aging changes in the cartilage matrix. Osteoarthr Cartil.

[124] Jeon OH, David N, Campisi J, Elisseeff JH (2018). Senescent cells and osteoarthritis: a painful connection. J Clin Invest.

[125] Frej F, Peter MN. Telomere biology and vascular aging. In: Early Vascular Aging (EVA). Elsevier; 2015. p. 201–11. https://www.sciencedirect.com/science/article/pii/B978012801387800020X. Accessed 1 Dec 2023.

[126] Hu MC, Shiizaki K, Kuro-o M, Moe OW (2013). Fibroblast growth factor 23 and Klotho: physiology and pathophysiology of an endocrine network of mineral metabolism. Annu Rev Physiol.

[127] Xu Y, Sun Z (2015). Molecular basis of Klotho: from gene to function in aging. Endocr Rev.

[128] Martins R, Lithgow GJ, Link W (2016). Long live FOXO : unraveling the role of FOXO proteins in aging and longevity. Aging Cell.

[129] Chen C, Zhou M, Ge Y, Wang X (2020). SIRT1 and aging related signaling pathways. Mech Ageing Dev.

[130] Korotkov A, Seluanov A, Gorbunova V (2021). Sirtuin 6: linking longevity with genome and epigenome stability. Trends Cell Biol.

[131] Tang M, Li Z, Zhang C, Lu X, Tu B, Cao Z (2019). SIRT7-mediated ATM deacetylation is essential for its deactivation and DNA damage repair. Sci Adv.

[132] Wang Y, Du L, Liang X, Meng P, Bi L, Wang Y (2019). Sirtuin 4 depletion promotes hepatocellular carcinoma tumorigenesis through regulating adenosine-monophosphate–activated protein kinase alpha/mammalian target of rapamycin axis in mice. Hepatology.

[133] Grunewald M, Kumar S, Sharife H, Volinsky E, Gileles-Hillel A, Licht T (2021). Counteracting age-related VEGF signaling insufficiency promotes healthy aging and extends life span. Science.

[134] Boone I, Tuerlings M, Coutinho de Almeida R, Lehmann J, Ramos Y, Nelissen R, et al. Identified senescence endotypes in aged cartilage are reflected in the blood metabolome. Geroscience. 2023; 10.1007/s11357-023-01001-2 PMC10828277 37962736

[135] Zarka M, Haÿ E, Cohen-Solal M (2022). YAP/TAZ in bone and cartilage biology. Front Cell Dev Biol.

[136] Horváth E, Sólyom Á, Székely J, Nagy EE, Popoviciu H (2023). Inflammatory and metabolic signaling interfaces of the hypertrophic and senescent chondrocyte phenotypes associated with osteoarthritis. Int J Mol Sci.

[137] Fu L, Hu Y, Song M, Liu Z, Zhang W, Yu FX (2019). Up-regulation of FOXD1 by YAP alleviates senescence and osteoarthritis. PLoS Biol..

[138] Ren X, Hu B, Song M, Ding Z, Dang Y, Liu Z (2019). Maintenance of nucleolar homeostasis by CBX4 alleviates senescence and osteoarthritis. Cell Rep.

[139] Jing Y, Jiang X, Ji Q, Wu Z, Wang W, Liu Z (2023). Genome-wide CRISPR activation screening in senescent cells reveals SOX5 as a driver and therapeutic target of rejuvenation. Cell Stem Cell.

[140] Yu J, Li T, Zhu J (2023). Gene therapy strategies targeting aging-related diseases. Aging Dis.

[141] Porter SN, Levine RM, Pruett-Miller SM (2019). A practical guide to genome editing using targeted nuclease technologies. Compr Physiol.

[142] Ousterout DG, Gersbach CA (2016). The development of TALE nucleases for biotechnology. Methods Mol Biol.

[143] Li HL, Nakano T, Hotta A (2014). Genetic correction using engineered nucleases for gene therapy applications. Dev Growth Differ.

[144] Varela-Eirín M, Varela-Vázquez A, Guitián-Caamaño A, Paíno CL, Mato V, Largo R (2018). Targeting of chondrocyte plasticity via connexin43 modulation attenuates cellular senescence and fosters a pro-regenerative environment in osteoarthritis. Cell Death Dis.

[145] Nettelbladt E, Sundblad L (1959). Protein patterns in synovial fluid and serum in rheumatoid arthritis and osteoarthritis. Arthritis Rheum.

[146] Robinson WH, Lepus CM, Wang Q, Raghu H, Mao R, Lindstrom TM (2016). Low-grade inflammation as a key mediator of the pathogenesis of osteoarthritis. Nat Rev Rheumatol.

[147] Liu S, Deng Z, Chen K, Jian S, Zhou F, Yang Y (2022). Cartilage tissue engineering: from proinflammatory and anti-inflammatory cytokines to osteoarthritis treatments (review). Mol Med Rep.

[148] Liu S, Cao C, Zhang Y, Liu G, Ren W, Ye Y (2019). PI3K/Akt inhibitor partly decreases TNF-α-induced activation of fibroblast-like synoviocytes in osteoarthritis. J Orthop Surg Res.

[149] Ding X, Zhang Y, Huang Y, Liu S, Lu H, Sun T (2015). Cadherin-11 involves in synovitis and increases the migratory and invasive capacity of fibroblast-like synoviocytes of osteoarthritis. Int Immunopharmacol.

[150] Chenoufi HL, Diamant M, Rieneck K, Lund B, Stein GS, Lian JB (2001). Increased mRNA expression and protein secretion of interleukin-6 in primary human osteoblasts differentiated in vitro from rheumatoid and osteoarthritic bone. J Cell Biochem.

[151] Liao Y, Ren Y, Luo X, Mirando AJ, Long JT, Leinroth A (2022). Interleukin-6 signaling mediates cartilage degradation and pain in posttraumatic osteoarthritis in a sex-specific manner. Sci Signal..

[152] Jenei-Lanzl Z, Meurer A, Zaucke F (2019). Interleukin-1β signaling in osteoarthritis—chondrocytes in focus. Cell Signal.

[153] Loeser RF, Erickson EA, Long DL (2008). Mitogen-activated protein kinases as therapeutic targets in osteoarthritis. Curr Opin Rheumatol.

[154] Zhou S, Lu W, Chen L, Ge Q, Chen D, Xu Z (2017). AMPK deficiency in chondrocytes accelerated the progression of instability-induced and ageing-associated osteoarthritis in adult mice. Sci Rep.

[155] Terkeltaub R, Yang B, Lotz M, Liu-Bryan R (2011). Chondrocyte AMP-activated protein kinase activity suppresses matrix degradation responses to proinflammatory cytokines interleukin-1β and tumor necrosis factor α. Arthritis Rheum.

[156] Motomura H, Seki S, Shiozawa S, Aikawa Y, Nogami M, Kimura T (2018). A selective c-Fos/AP-1 inhibitor prevents cartilage destruction and subsequent osteophyte formation. Biochem Biophys Res Commun.

[157] Wu H, Xu T, Chen Z, Wang Y, Li K, Chen PS (2020). Specific inhibition of FAK signaling attenuates subchondral bone deterioration and articular cartilage degeneration during osteoarthritis pathogenesis. J Cell Physiol.

[158] Yanoshita M, Hirose N, Okamoto Y, Sumi C, Takano M, Nishiyama S (2018). Cyclic tensile strain upregulates pro-inflammatory cytokine expression via FAK-MAPK signaling in chondrocytes. Inflammation..

[159] Sang F, Xu J, Chen Z, Liu Q, Jiang W (2021). Low-intensity pulsed ultrasound alleviates osteoarthritis condition through focal adhesion kinase-mediated chondrocyte proliferation and differentiation. Cartilage.

[160] Miyauchi A, Kim-Kaneyama JR, Lei XF, Chang SH, Saito T, Haraguchi S (2019). Alleviation of murine osteoarthritis by deletion of the focal adhesion mechanosensitive adapter, Hic-5. Sci Rep.

[161] Miyauchi A, Noguchi M, Lei XF, Sakaki M, Kobayashi-Tanabe M, Haraguchi S (2023). Knockdown of mechanosensitive adaptor Hic-5 ameliorates post-traumatic osteoarthritis in rats through repression of MMP-13. Sci Rep.

[162] Chen S, He T, Zhong Y, Chen M, Yao Q, Chen D (2023). Roles of focal adhesion proteins in skeleton and diseases. Acta Pharm Sin B.

[163] Loeser RF (2014). Integrins and chondrocyte-matrix interactions in articular cartilage. Matrix Biol.

[164] Zhao X, Petursson F, Viollet B, Lotz M, Terkeltaub R, Liu-Bryan R (2014). Peroxisome proliferator-activated receptor γ coactivator 1α and FoxO3A mediate chondroprotection by AMP-activated protein kinase. Arthritis Rheumatol.

[165] Wang F, Wang Q, Zhu M, Sun Q (2020). Foxo3a aggravates inflammation and induces apoptosis in IL-1-treated rabbit chondrocytes via positively regulating tenascin-c. Folia Histochem Cytobiol.

[166] Nummenmaa E, Hämäläinen M, Moilanen T, Vuolteenaho K, Moilanen E (2015). Effects of FGF-2 and FGF receptor antagonists on MMP enzymes, aggrecan, and type II collagen in primary human OA chondrocytes. Scand J Rheumatol.

[167] Rabie MA, Sayed RH, Venkatesan JK, Madry H, Cucchiarini M, El Sayed NS (2023). Intra-articular injection of rAAV-hFGF-2 ameliorates monosodium iodoacetate-induced osteoarthritis in rats via inhibiting TLR-4 signaling and activating TIMP-1. Toxicol Appl Pharmacol.

[168] Boehme KA, Rolauffs B (2018). Onset and progression of human osteoarthritis-can growth factors, inflammatory cytokines, or differential miRNA expression concomitantly induce proliferation, ECM degradation, and inflammation in articular cartilage?. Int J Mol Sci.

[169] Chen TM, Chen YH, Sun HS, Tsai SJ (2019). Fibroblast growth factors: potential novel targets for regenerative therapy of osteoarthritis. Chin J Physiol.

[170] Zeng CY, Wang XF, Hua FZ (2022). HIF-1α in osteoarthritis: from pathogenesis to therapeutic implications. Front Pharmacol.

[171] Zhang XA, Kong H (2023). Mechanism of HIFs in osteoarthritis. Front Immunol.

[172] Yang S, Kim J, Ryu JH, Oh H, Chun CH, Kim BJ (2010). Hypoxia-inducible factor-2alpha is a catabolic regulator of osteoarthritic cartilage destruction. Nat Med.

[173] Saito T, Kawaguchi H (2010). HIF-2α as a possible therapeutic target of osteoarthritis. Osteoarthritis Cartilage.

[174] Deng Y, Lu J, Li W, Wu A, Zhang X, Tong W (2018). Reciprocal inhibition of YAP/TAZ and NF-κB regulates osteoarthritic cartilage degradation. Nat Commun.

[175] Hao X, Zhao J, Jia L, He T, Wang H, Fan J (2022). XMU-MP-1 attenuates osteoarthritis via inhibiting cartilage degradation and chondrocyte apoptosis. Front Bioeng Biotechnol.

[176] Zhou Q, Ren Q, Jiao L, Huang J, Yi J, Chen J (2022). The potential roles of JAK/STAT signaling in the progression of osteoarthritis. Front Endocrinol (Lausanne).

[177] Malemud CJ (2017). Negative regulators of JAK/STAT signaling in rheumatoid arthritis and osteoarthritis. Int J Mol Sci.

[178] Sun K, Luo J, Guo J, Yao X, Jing X, Guo F (2020). The PI3K/AKT/mTOR signaling pathway in osteoarthritis: a narrative review. Osteoarthr Cartil.

[179] Caramés B, Hasegawa A, Taniguchi N, Miyaki S, Blanco FJ, Lotz M (2012). Autophagy activation by rapamycin reduces severity of experimental osteoarthritis. Ann Rheum Dis.

[180] Kobayashi H, Hirata M, Saito T, Itoh S, Chung UI, Kawaguchi H (2013). Transcriptional induction of ADAMTS5 protein by nuclear factor-κB (NF-κB) family member RelA/p65 in chondrocytes during osteoarthritis development. J Biol Chem.

[181] Choi MC, Jo J, Park J, Kang HK, Park Y (2019). NF-κB signaling pathways in osteoarthritic cartilage destruction. Cells.

[182] Raymond L, Eck S, Hays E, Tomek I, Kantor S, Vincenti M (2007). RelA is required for IL-1beta stimulation of matrix metalloproteinase-1 expression in chondrocytes. Osteoarthr Cartil.

[183] Goldring MB, Otero M (2011). Inflammation in osteoarthritis. Curr Opin Rheumatol.

[184] Lee WS, Yasuda S, Kono M, Kudo Y, Shimamura S, Kono M (2020). MicroRNA-9 ameliorates destructive arthritis through down-regulation of NF-κB1-RANKL pathway in fibroblast-like synoviocytes. Clin Immunol.

[185] Tang S, Nie X, Ruan J, Cao Y, Kang J, Ding C (2022). Circular RNA circNFKB1 promotes osteoarthritis progression through interacting with ENO1 and sustaining NF-κB signaling. Cell Death Dis.

[186] De Bosscher K, Vanden Berghe W, Vermeulen L, Plaisance S, Boone E, Haegeman G (2000). Glucocorticoids repress NF-kappaB-driven genes by disturbing the interaction of p65 with the basal transcription machinery, irrespective of coactivator levels in the cell. Proc Natl Acad Sci U S A.

[187] Arra M, Swarnkar G, Ke K, Otero JE, Ying J, Duan X (2020). LDHA-mediated ROS generation in chondrocytes is a potential therapeutic target for osteoarthritis. Nat Commun.

[188] Olivotto E, Otero M, Marcu KB, Goldring MB (2015). Pathophysiology of osteoarthritis: canonical NF-κB/IKKβ-dependent and kinase-independent effects of IKKα in cartilage degradation and chondrocyte differentiation. RMD Open.

[189] Chen YL, Yan DY, Wu CY, Xuan JW, Jin CQ, Hu XL (2021). Maslinic acid prevents IL-1β-induced inflammatory response in osteoarthritis via PI3K/AKT/NF-κB pathways. J Cell Physiol.

[190] Marcu KB, Otero M, Olivotto E, Borzi RM, Goldring MB (2010). NF-kappaB signaling: multiple angles to target OA. Curr Drug Targets..

[191] Wondimu EB, Culley KL, Quinn J, Chang J, Dragomir CL, Plumb DA (2018). Elf3 contributes to cartilage degradation in vivo in a surgical model of post-traumatic osteoarthritis. Sci Rep.

[192] Zheng T, Li Y, Zhang X, Xu J, Luo M (2022). Exosomes derived from miR-212-5p overexpressed human synovial mesenchymal stem cells suppress chondrocyte degeneration and inflammation by targeting ELF3. Front Bioeng Biotechnol.

[193] Liu Z, Chen J, Mirando AJ, Wang C, Zuscik MJ, O’Keefe RJ (2015). A dual role for NOTCH signaling in joint cartilage maintenance and osteoarthritis. Sci Signal..

[194] Cheng HJ, Hsu WT, Chen CN, Li C (2020). Activation of NOTCH1 by shear force elicits immediate cytokine expression in human chondrocytes. Int J Mol Sci.

[195] Sheng W, Wang Q, Qin H, Cao S, Wei Y, Weng J (2023). Osteoarthritis: role of peroxisome proliferator-activated receptors. Int J Mol Sci.

[196] Fahmi H, Martel-Pelletier J, Pelletier JP, Kapoor M (2011). Peroxisome proliferator-activated receptor gamma in osteoarthritis. Mod Rheumatol.

[197] Jouzeau JY, Moulin D, Koufany M, Sebillaud S, Bianchi A, Netter P (2008). Pathophysiological relevance of peroxisome proliferators activated receptors (PPAR) to joint diseases—the pro and con of agonists. J Soc Biol.

[198] Wang H, Su J, Yu M, Xia Y, Wei Y (2023). PGC-1α in osteoarthritic chondrocytes: from mechanism to target of action. Front Pharmacol.

[199] Matsuzaki T, Matsushita T, Takayama K, Matsumoto T, Nishida K, Kuroda R (2014). Disruption of Sirt1 in chondrocytes causes accelerated progression of osteoarthritis under mechanical stress and during ageing in mice. Ann Rheum Dis.

[200] Liu-Bryan R (2015). Inflammation and intracellular metabolism: new targets in OA. Osteoarthr Cartil.

[201] Chen Y, Wu YY, Si HB, Lu YR, Shen B (2021). Mechanistic insights into AMPK-SIRT3 positive feedback loop-mediated chondrocyte mitochondrial quality control in osteoarthritis pathogenesis. Pharmacol Res.

[202] Xu K, He Y, Moqbel SAA, Zhou X, Wu L, Bao J (2021). SIRT3 ameliorates osteoarthritis via regulating chondrocyte autophagy and apoptosis through the PI3K/Akt/mTOR pathway. Int J Biol Macromol.

[203] Chen J, Chen S, Cai D, Wang Q, Qin J (2022). The role of Sirt6 in osteoarthritis and its effect on macrophage polarization. Bioengineered.

[204] Blaney Davidson EN, Van Caam APM, Van Der Kraan PM (2017). Osteoarthritis year in review 2016: biology. Osteoarthr Cartil.

[205] Ailixiding M, Aibibula Z, Iwata M, Piao J, Hara Y, Koga D (2015). Pivotal role of Sirt6 in the crosstalk among ageing, metabolic syndrome and osteoarthritis. Biochem Biophys Res Commun.

[206] Cheng J, Hu X, Dai L, Zhang X, Ren B, Shi W (2016). Inhibition of transforming growth factor β-activated kinase 1 prevents inflammation-related cartilage degradation in osteoarthritis. Sci Rep.

[207] Fechtner S, Fox DA, Ahmed S (2017). Transforming growth factor β activated kinase 1: a potential therapeutic target for rheumatic diseases. Rheumatology (Oxford).

[208] Chen Q, Wu S, Wu Y, Chen L, Pang Q (2018). MiR-149 suppresses the inflammatory response of chondrocytes in osteoarthritis by down-regulating the activation of TAK1/NF-κB. Biomed Pharmacother.

[209] Gamer LW, Pregizer S, Gamer J, Feigenson M, Ionescu A, Li Q (2018). The Role of Bmp2 in the maturation and maintenance of the murine knee joint. J Bone Miner Res.

[210] Goldring MB, Otero M, Tsuchimochi K, Ijiri K, Li Y (2008). Defining the roles of inflammatory and anabolic cytokines in cartilage metabolism. Ann Rheum Dis.

[211] Thielen NGM, van der Kraan PM, van Caam APM (2019). TGFβ/BMP signaling pathway in cartilage homeostasis. Cells.

[212] Boon MR, Van Der Horst G, Van Der Pluijm G, Tamsma JT, Smit JWA, Rensen PCN (2011). Bone morphogenetic protein 7: A broad-spectrum growth factor with multiple target therapeutic potency. Cytokine Growth Factor Rev.

[213] Takahashi T, Muneta T, Tsuji K, Sekiya I (2011). BMP-7 inhibits cartilage degeneration through suppression of inflammation in rat zymosan-induced arthritis. Cell Tissue Res.

[214] Yoo KH, Thapa N, Chwae YJ, Yoon SH, Kim BJ, Lee JO (2022). Transforming growth factor-β family and stem cell-derived exosome therapeutic treatment in osteoarthritis (Review). Int J Mol Med.

[215] Wiegertjes R, van Caam A, van Beuningen H, Koenders M, van Lent P, van der Kraan P (2019). TGF-β dampens IL-6 signaling in articular chondrocytes by decreasing IL-6 receptor expression. Osteoarthr Cartil.

[216] Barreto G, Manninen M, Eklund K (2020). Osteoarthritis and toll-like receptors: when innate immunity meets chondrocyte apoptosis. Biology (Basel)..

[217] Cheng J, Li M, Bai R (2022). The Wnt signaling cascade in the pathogenesis of osteoarthritis and related promising treatment strategies. Front Physiol.

[218] Nakamura Y, Nawata M, Wakitani S (2005). Expression profiles and functional analyses of Wnt-related genes in human joint disorders. Am J Pathol.

[219] Lin J, Jia S, Zhang W, Nian M, Liu P, Yang L (2023). Recent advances in small molecule inhibitors for the treatment of osteoarthritis. J Clin Med.

[220] Karlsen TA, Pernas PF, Staerk J, Caglayan S, Brinchmann JE (2016). Generation of IL1β-resistant chondrocytes using CRISPR-CAS genome editing. Osteoarthr Cartil.

[221] Dooley C, Murphy M (2021). Using CRISPR/Cas9 gene editting systems to understand the function of interleukin 16 in progression of osteoarthritis. Osteoarthr Cartil.

[222] Bonato A, Fisch P, Ponta S, Fercher D, Manninen M, Weber D (2023). Engineering inflammation-resistant cartilage: bridging gene therapy and tissue engineering. Adv Healthc Mater.

[223] Klimak M, Nims RJ, Pferdehirt L, Collins KH, Harasymowicz NS, Oswald SJ (2021). Immunoengineering the next generation of arthritis therapies. Acta Biomater.

[224] Gerace D, Martiniello-Wilks R, Nassif NT, Lal S, Steptoe R, Simpson AM (2017). CRISPR-targeted genome editing of mesenchymal stem cell-derived therapies for type 1 diabetes: a path to clinical success?. Stem Cell Res Ther.

[225] Pawitan JA, Bui TA, Mubarok W, Antarianto RD, Nurhayati RW, Dilogo IH (2020). Enhancement of the therapeutic capacity of mesenchymal stem cells by genetic modification: a systematic review. Front Cell Dev Biol.

[226] Brunger JM, Zutshi A, Willard VP, Gersbach CA, Guilak F (2017). CRISPR/Cas9 editing of murine induced pluripotent stem cells for engineering inflammation-resistant tissues. Arthritis Rheumatol.

[227] Brunger JM, Zutshi A, Willard VP, Gersbach CA, Guilak F (2017). Genome engineering of stem cells for autonomously regulated, closed-loop delivery of biologic drugs. Stem Cell Rep.

[228] Choi YR, Collins KH, Springer LE, Pferdehirt L, Ross AK, Wu CL (2021). A genome-engineered bioartificial implant for autoregulated anticytokine drug delivery. Sci Adv.

[229] Safari F, Farajnia S, Arya M, Zarredar H, Nasrolahi A (2018). CRISPR and personalized Treg therapy: new insights into the treatment of rheumatoid arthritis. Immunopharmacol Immunotoxicol.

[230] Choi YR, Collins KH, Lee JW, Kang HJ, Guilak F (2019). Genome engineering for osteoarthritis: from designer cells to disease-modifying drugs. Tissue Eng Regen Med.

[231] Makris EA, Gomoll AH, Malizos KN, Hu JC, Athanasiou KA (2015). Repair and tissue engineering techniques for articular cartilage. Nat Rev Rheumatol.

[232] Vlashi R, Zhang X, Li H, Chen G (2024). Potential therapeutic strategies for osteoarthritis via CRISPR/Cas9 mediated gene editing. Rev Endocr Metab Disord..

[233] Lories RJ, Luyten FP (2011). The bone-cartilage unit in osteoarthritis. Nat Rev Rheumatol.

[234] Grath A, Dai G (2019). Direct cell reprogramming for tissue engineering and regenerative medicine. J Biol Eng.

[235] Pittenger MF, Mackay AM, Beck SC, Jaiswal RK, Douglas R, Mosca JD (1999). Multilineage potential of adult human mesenchymal stem cells. Science.

[236] Bartholomew A, Sturgeon C, Siatskas M, Ferrer K, McIntosh K, Patil S (2002). Mesenchymal stem cells suppress lymphocyte proliferation in vitro and prolong skin graft survival in vivo. Exp Hematol.

[237] Almalki SG, Agrawal DK (2016). Key transcription factors in the differentiation of mesenchymal stem cells. Differentiation.

[238] Barrero MJ, Boué S, Belmonte JCI (2010). Epigenetic mechanisms that regulate cell identity. Cell Stem Cell.

[239] Chen Q, Shou P, Zheng C, Jiang M, Cao G, Yang Q (2016). Fate decision of mesenchymal stem cells: adipocytes or osteoblasts?. Cell Death Differ.

[240] Wu CC, Liu FL, Sytwu HK, Tsai CY, Chang DM (2016). CD146+ mesenchymal stem cells display greater therapeutic potential than CD146– cells for treating collagen-induced arthritis in mice. Stem Cell Res Ther.

[241] Augello A, De Bari C (2010). The regulation of differentiation in mesenchymal stem cells. Hum Gene Ther.

[242] Filho DM, de Carvalho RP, Oliveira LF, Dos Santos ALRT, Parreira RC, Pinto MCX (2019). Enhancing the therapeutic potential of mesenchymal stem cells with the CRISPR-Cas system. Stem Cell Rev Rep.

[243] Kwon DY, Zhao YT, Lamonica JM, Zhou Z (2017). Locus-specific histone deacetylation using a synthetic CRISPR-Cas9-based HDAC. Nat Commun.

[244] Liu XS, Wu H, Ji X, Stelzer Y, Wu X, Czauderna S (2016). Editing DNA methylation in the mammalian genome. Cell.

[245] Chaudhry N, Muhammad H, Seidl C, Downes D, Young DA, Hao Y (2022). Highly efficient CRISPR-Cas9-mediated editing identifies novel mechanosensitive microRNA-140 targets in primary human articular chondrocytes. Osteoarthr Cartil.

[246] Seidl CI, Fulga TA, Murphy CL (2019). CRISPR-Cas9 targeting of MMP13 in human chondrocytes leads to significantly reduced levels of the metalloproteinase and enhanced type II collagen accumulation. Osteoarthr Cartil.

[247] Yue K, Trujillo-de Santiago G, Alvarez MM, Tamayol A, Annabi N, Khademhosseini A (2015). Synthesis, properties, and biomedical applications of gelatin methacryloyl (GelMA) hydrogels. Biomaterials.

[248] Farhang N, Davis B, Weston J, Ginley-Hidinger M, Gertz J, Bowles RD (2020). Synergistic CRISPRa-regulated chondrogenic extracellular matrix deposition without exogenous growth factors. Tissue Eng Part A.

[249] Nonaka K, Han X, Kato H, Sato H, Yamaza H, Hirofuji Y (2019). Novel gain-of-function mutation of TRPV4 associated with accelerated chondrogenic differentiation of dental pulp stem cells derived from a patient with metatropic dysplasia. Biochem Biophys Rep.

[250] Zhong G, Madry H, Cucchiarini M (2022). Mitochondrial genome editing to treat human osteoarthritis—a narrative review. Int J Mol Sci.

[251] Maneiro E, Martín MA, De Andres MC, López-Armada MJ, Fernández-Sueiro JL, Del Hoyo P (2003). Mitochondrial respiratory activity is altered in osteoarthritic human articular chondrocytes. Arthritis Rheum.

[252] Blanco FJ, Rego I, Ruiz-Romero C (2011). The role of mitochondria in osteoarthritis. Nat Rev Rheumatol.

[253] Ruiz-Romero C, Calamia V, Mateos J, Carreira V, Martiénez-Gomariz M, Fernaéndez M (2009). Mitochondrial dysregulation of osteoarthritic human articular chondrocytes analyzed by proteomics: a decrease in mitochondrial superoxide dismutase points to a redox imbalance. Mol Cell Proteomics.

[254] Blanco FJ, Valdes AM, Rego-Pérez I (2018). Mitochondrial DNA variation and the pathogenesis of osteoarthritis phenotypes. Nat Rev Rheumatol.

[255] Taanman JW (1999). The mitochondrial genome: structure, transcription, translation and replication. Biochim Biophys Acta BBA Bioenerget..

[256] Fontana GA, Gahlon HL (2020). Mechanisms of replication and repair in mitochondrial DNA deletion formation. Nucleic Acids Res.

[257] Larsen NB, Rasmussen M, Rasmussen LJ (2005). Nuclear and mitochondrial DNA repair: similar pathways?. Mitochondrion.

[258] Bolduc JA, Collins JA, Loeser RF (2019). Reactive oxygen species, aging and articular cartilage homeostasis. Free Radic Biol Med.

[259] Gorman GS, Chinnery PF, DiMauro S, Hirano M, Koga Y, McFarland R (2016). Mitochondrial diseases. Nat Rev Dis Primers.

[260] Muratovska A, Lightowlers RN, Taylor RW, Turnbull DM, Smith RA, Wilce JA (2001). Targeting peptide nucleic acid (PNA) oligomers to mitochondria within cells by conjugation to lipophilic cations: implications for mitochondrial DNA replication, expression and disease. Nucleic Acids Res.

[261] Chinnery PF, Taylor RW, Diekert K, Lill R, Turnbull DM, Lightowlers RN (1999). Peptide nucleic acid delivery to human mitochondria. Gene Ther.

[262] Taylor RW, Wardell TM, Smith PM, Muratovska A, Murphy MP, Turnbull DM (2001). An antigenomic strategy for treating heteroplasmic mtDNA disorders. Adv Drug Deliv Rev.

[263] Bacman SR, Williams SL, Garcia S, Moraes CT (2010). Organ-specific shifts in mtDNA heteroplasmy following systemic delivery of a mitochondria-targeted restriction endonuclease. Gene Ther.

[264] Gammage PA, Rorbach J, Vincent AI, Rebar EJ, Minczuk M (2014). Mitochondrially targeted ZFNs for selective degradation of pathogenic mitochondrial genomes bearing large-scale deletions or point mutations. EMBO Mol Med.

[265] Gammage PA, Gaude E, Van Haute L, Rebelo-Guiomar P, Jackson CB, Rorbach J (2016). Near-complete elimination of mutant mtDNA by iterative or dynamic dose-controlled treatment with mtZFNs. Nucleic Acids Res.

[266] Jo A, Ham S, Lee GH, Lee YI, Kim S, Lee YS (2015). Efficient mitochondrial genome editing by CRISPR/Cas9. Biomed Res Int.

[267] Gammage PA, Moraes CT, Minczuk M (2018). Mitochondrial genome engineering: the revolution may not be CRISPR-Ized. Trends Genet.

